# Long-Term Quiescent Fibroblast Cells Transit into Senescence

**DOI:** 10.1371/journal.pone.0115597

**Published:** 2014-12-22

**Authors:** Shiva Marthandan, Steffen Priebe, Peter Hemmerich, Karolin Klement, Stephan Diekmann

**Affiliations:** 1 Leibniz-Institute for Age Research- Fritz Lipmann Institute, JenAge (Jena Centre for Systems Biology of Aging), Beutenbergstrasse 11, Jena, Germany; 2 Leibniz Institute for Natural Product Research and Infection Biology - Hans-Knöll-Institute e.V. (HKI), Jena, Germany; German Cancer Research Center, Germany

## Abstract

Cellular senescence is described to be a consequence of telomere erosion during the replicative life span of primary human cells. Quiescence should therefore not contribute to cellular aging but rather extend lifespan. Here we tested this hypothesis and demonstrate that cultured long-term quiescent human fibroblasts transit into senescence due to similar cellular mechanisms with similar dynamics and with a similar maximum life span as proliferating controls, even under physiological oxygen conditions. Both, long-term quiescent and senescent fibroblasts almost completely fail to undergo apoptosis. The transition of long-term quiescent fibroblasts into senescence is also independent of HES1 which protects short-term quiescent cells from becoming senescent. Most significantly, DNA damage accumulates during senescence as well as during long-term quiescence at physiological oxygen levels. We suggest that telomere-independent, potentially maintenance driven gradual induction of cellular senescence during quiescence is a counterbalance to tumor development.

## Introduction

Many cells within our bodies, including fibroblasts, hepatocytes, lymphocytes, stem cells and germ cells, are in the state of quiescence, defined as a reversible cell cycle arrest with temporary absence of proliferation [1]. Pathologies associated with quiescence include auto-immune diseases, fibrosis, and chronic wounds. Some of these cells maintain a quiescent state for long periods of time, even years, and quiescent cells are defined to retain the ability to return into the cell cycle. *In vivo*, quiescence is considered to limit the uncontrolled proliferation of cells, especially stem cells, whose proliferation has to be controlled properly in order to maintain tissue function, therefore contributing to tissue homeostasis [2]–[6]. A number of functional changes have been associated with quiescence including modified metabolism [7]–[9] and altered chromatin conformation [10]–[12]. Quiescence is not a passive default state, but instead is actively maintained by specific molecular mechanisms [13], [14]. Human diploid fibroblasts can enter quiescence in response to signals including loss of adhesion, contact inhibition, and mitogen withdrawal. Each of these anti-proliferative signals induce a major induction-specific reprogramming of gene expression, either enforcing the non-dividing state by regulating genes involved in cell division, or ensuring the reversibility of quiescence by protecting cells from damage induced by free radicals, while other changes indicate the involvement in pathways protecting quiescent cells against transition into terminal differentiation [15]. Thus, quiescence is a collection of states determined by the initiating signal; however, a number of genes are universally characteristic of quiescence, implying the existence of a genetic program of quiescence common to the different quiescent states [15].

Quiescent cells show low expression of cyclins and cyclin dependent kinases (CDKs) [6], [16], [17] as well as of the CDK inhibitors (CDKIs) p19 or p16 [1], [18] but high expression of CDKIs p21, p27, p53 and p57 [2]. Up-regulation of p21 occurs during several cell cycle arrested states, including quiescence, senescence and terminal differentiation [19]–[22], and is mostly accompanied by expression of p27 [18], [23]–[26]. Quiescence can easily be reversed by depletion of p21 [1], and, *vice versa*, cells with depleted p21 show impaired entry into quiescence. Quiescence is not simply a downstream consequence of cell cycle exit. Specific inhibition of CDKs arrests the cell cycle, but this neither induces the quiescence-specific gene expression program nor resistance to terminal differentiation [15]. Thus, the quiescence program of gene expression, but not direct CDK inhibition, ensures the reversibility of the quiescence state. Due to the up-regulation of p21, quiescent cells are endangered to transit into senescence. Cells having been quiescent for 10 days are protected against this transition into senescence by the up-regulation of the transcriptional repressor HES1 [27].

In order to be reversible, quiescence must grant the return into the cell cycle. Consequently, quiescent cells repress transition into terminal differentiation in which cell cycle arrest is irreversible [15]. However, when transition into irreversible cell cycle arrest is suppressed, reversible non-dividing quiescent cells are less protected against cancer development and are subject to tumor development. While short-term quiescent cells were described to be protected against transition into senescence [27], long-term quiescent cells may protect themselves against malignant transformation by implementing a senescence-associated cell cycle arrest over longer periods of time. Indeed, most of a human foetal skin fibroblast cell population while being long-term quiescent, were observed to transit into senescence [28]. This however violates the definition of a quiescent cell population of being able, even after years of quiescence, to completely return into the cell cycle. Here we resolve this contradiction by showing that long-term quiescent primary human cultured fibroblast MRC-5 and WI-38 cells transit into senescence. It remains to be shown to what extent these findings, observed for cultured cells, also hold for cells in tissue (*in vivo*). After months of quiescence, MRC-5 and WI-38 cells still display HES1 up-regulation, but this is unable to protect the cells against transition into senescence. Only a few percent of the cells in the population are able to return into the cell cycle.

Cellular senescence is a cell cycle arrested state in which normal diploid primary cells have lost their proliferative potential [29]. Senescence is regarded as a tumor suppressor mechanism at young age but a contributor to tissue aging later in life (“antagonistic pleiotropy”), [30]. *In vivo*, cellular senescence is thought to significantly contribute to the aging process [31]–[35]. Senescence displays a specific phenotype, including a flattened and enlarged cellular morphology [36], [37], an increased activity of senescence-associated β-galactosidase (SA-β Gal) activity [38], and expression of additional, more or less specific, molecular signatures. Premature senescence is supposed to be mainly mediated by an increased and persistent DNA damage response resulting from genotoxic stress [34], [39]–[49]. In particular, replicative senescence can be mediated by telomere shortening and a subsequent persistent DNA damage response from unprotected telomeres [36], [50]–[53]. Permanent senescence-associated cell cycle arrest is initiated and maintained by the p53-p21 and p16-pRb pathways [54], [55]. Cellular senescence can be reversed when maintained only by p53-p21 induction [1], [19], [56], [57]. RNAi-mediated depletion of p21 but not p16 leads to cell cycle re-entry of senescent keratinocytes [1]. Suppression of the p16 pathway might lead to S-phase re-entry and replication but not cell division [57], [58]. Therefore, both p21 and p16 driven pathways constitute important mechanism to ensure the irreversibility of cellular senescence.

Telomere shortening as a basic concept for aging assumes that each successive cell division acts as a mitotic counting mechanism inducing replicative senescence [59]–[63]. According to this concept, induction of quiescence for a defined amount of time would be predicted to prolong the lifespan of fibroblasts in comparison to constantly proliferating cells. In contrast to this prediction, after long-term quiescence primary human foreskin fibroblasts (HFF) were observed to transit into senescence despite of negligible telomere shortening [28], questioning that cell division and telomeric attrition is necessarily required for senescence [64]–[67]. Here we detect that during long-term quiescence also other human fibroblasts enter senescence. Thus, other effects than telomere shortening, like oxidative stress induced DNA damage, may be responsible for this transition [67]. This is supported by the fact that mouse fibroblasts senesce in culture although mice have very long telomeres. We therefore analyzed the transition of quiescent human primary MRC-5 and WI-38 fibroblast cells into senescence and apoptosis. We reduced the oxidative stress and found that WI-38 cells did not respond at all to this stress reduction, and MRC-5 cells only to a small amount. Our results suggest that other mechanisms beyond telomere shortening and oxidative stress drive human fibroblasts into senescence.

## Results

### Short periods of quiescence delay fibroblast aging but do not extend life span

**Figure 1:**
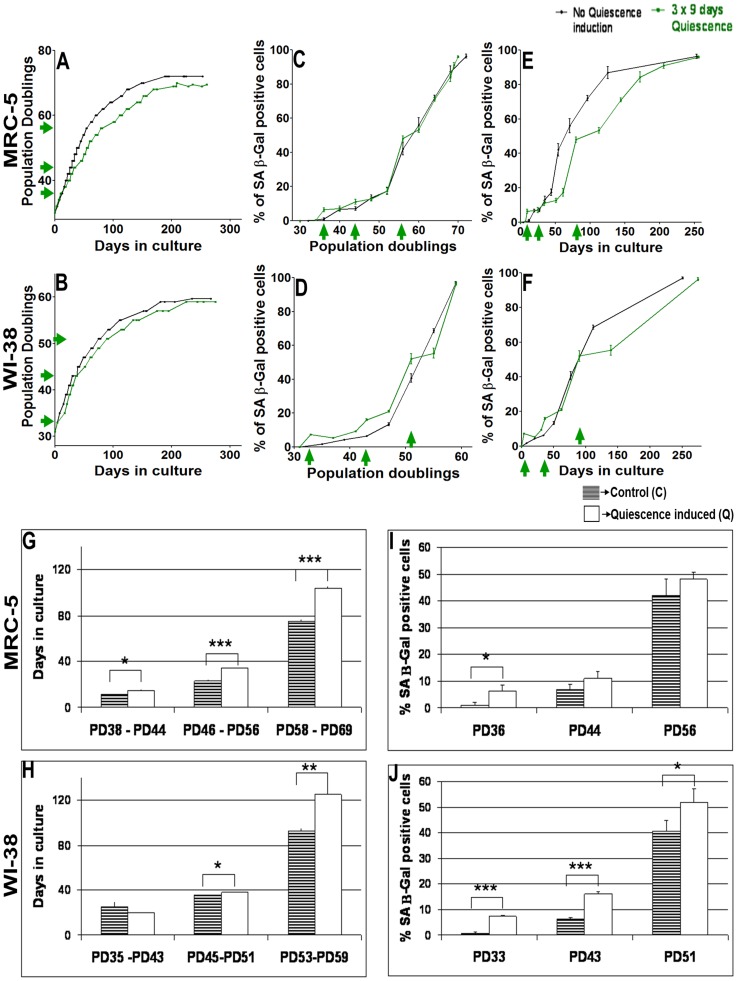
Impact of short term quiescence induction (3×9 days) in MRC-5 and WI-38 fibroblasts. (**A & B**) Growth curve of 2 independent MRC-5 (A) and WI-38 (B) fibroblast cell lines (control with no quiescence induction and a cell line where quiescence was induced 3 times separately for a span of 9 days by contact inhibition) maintained in culture at 20% O_2_ as triplicates from an early PD until senescence at late PDs. Each growth curve is measured in triplicate. Data points of all measurements are displayed (not the mean). (**B, C, D, E & F**) Percentage of SA-β gal positive cells at different time points of their growth in culture in the control MRC-5 (C & E) and WI-38 (D & F) fibroblast cell line and in the cell line where quiescence was induced 3 times separately. Fig. 1 C and D are plotted with PDs, whereas Fig. 1 E and F are plotted with days in culture in the y-axis. Each curve is measured in triplicate, the mean value is displayed with error bar (± S.E). (**G & H**) Quiescence was induced by contact inhibition in short periods of 9 days at 3 stages of the lifespan of MRC-5 (at PDs = 36, 44, 56) and WI-38 (at PDs = 33, 43, 51) fibroblasts maintained in culture at 20% O_2_. The plot shows the number of days spent by MRC-5 (G) and WI-38 (H) fibroblasts in culture between PDs 38 and 44, 46 and 56, and 58 and 69 for MRC-5 (G) and between PDs 35 and 43, 45 and 51, and 53 and 59 for WI-38 (H) for cells having been repeatedly quiescent compared to the control fibroblasts. (**I & J**) Percentage of SA-β gal positive cells at PD immediately after quiescence induction compared to their respective non-quiescence induced MRC-5 (I) and WI-38 (J) controls. The bars indicate the mean ± S.D. Values statistically different from their controls (t-test) are indicated with an asterix: * p<0.05, ** p<0.01, *** p<0.001. n = 3

In a first set of experiments, quiescence was induced (three times for a period of 9 days each) by contact inhibition in replicatively aging MRC-5 fibroblasts (at population doublings (PDs) 36, 44 and 56) or WI-38 cells (at PDs 33, 43, and 51) (Fig. 1). Contact inhibition as the quiescence inducing signal was selected because it keeps the quiescent cells in the same serum condition as the proliferating control cells, and thus allows for a direct comparison of cellular and molecular signatures. Nevertheless, we performed similar quiescence induction experiments implying serum starvation over 8 days, which yielded similar results. Cells resumed proliferation after each release from quiescence (Fig. 1 A and B). Constantly proliferating control MRC-5 or WI-38 fibroblasts reached maximum PDs of 72 or 61, respectively, whereas cells subjected to pulses of quiescence reached 69 PDs (MRC-5) or 59 PDs (WI-38). Thus, repeated short-term quiescence induction did not result in an increased proliferative potential of MRC-5 or WI-38 cells; rather, we observed a slightly reduced number of cell divisions in quiescence-pulsed cell cultures. The periods of quiescence had an influence on cellular behavior: after periods of quiescence, between same PD values MRC-5 and WI-38 fibroblast cells grew detectably slower compared to constantly proliferating control fibroblast cells (Fig. 1 A and B). A quantitative analysis revealed, compared to control cells, a significantly decreased growth rate of MRC-5 or WI-38 cells having been quiescent for three 9-day periods, but not after one period (Fig. 1 G and H). The increase of the amount of SA-β Gal positive cells was similar in both populations when being plotted versus PDs (Fig. 1 C and D), with the time-limited exception of the single time points at the end of the quiescence periods. An increase of β Gal after confluency-induced quiescence has been observed before [38], [68]–[72]. This effect is stronger in WI-38 compared to MRC-5 cells (Fig. 1 C, D, I and J). Plotting the SA-β Gal activity versus time in culture revealed delayed aging in MRC-5 cells after two and three 9-day periods of quiescence (Fig. 1 E) while for WI-38 cells this effect was observed only after three periods of quiescence (Fig. 1 F). Short-term (9 days) quiescence induction also resulted in a significant increase of anti-apoptotic protein Bcl-2 in both MRC-5 and WI-38 fibroblasts (data not shown), in agreement with previous studies [73], [74].

In cells having experienced periods of quiescence, p21 was up-regulated (S1 and S2 Figs.) [27] while p16 was reduced (S1 and S2 Figs., with the exception of WI-38 at PD 33) compared to proliferating controls. We detected only insignificant differences in expression levels of cyclin D1 (S1 and S2 Figs.) and D2 (S1 and S2 Figs.) and the DNA damage marker γH2A.X (S1 and S2 Figs.) between cells having experienced periods of quiescence and control cells (S1 and S2 Figs.).

Taken together, we observed that the longer times in culture of the cells having been in quiescence (3 times 9 days) compared to control cells, influence cellular properties. Next, we studied MRC-5 and WI-38 cells during and after longer periods of quiescence.

### Long-term quiescence of human fibroblasts resulted in senescence induction

**Figure 2:**
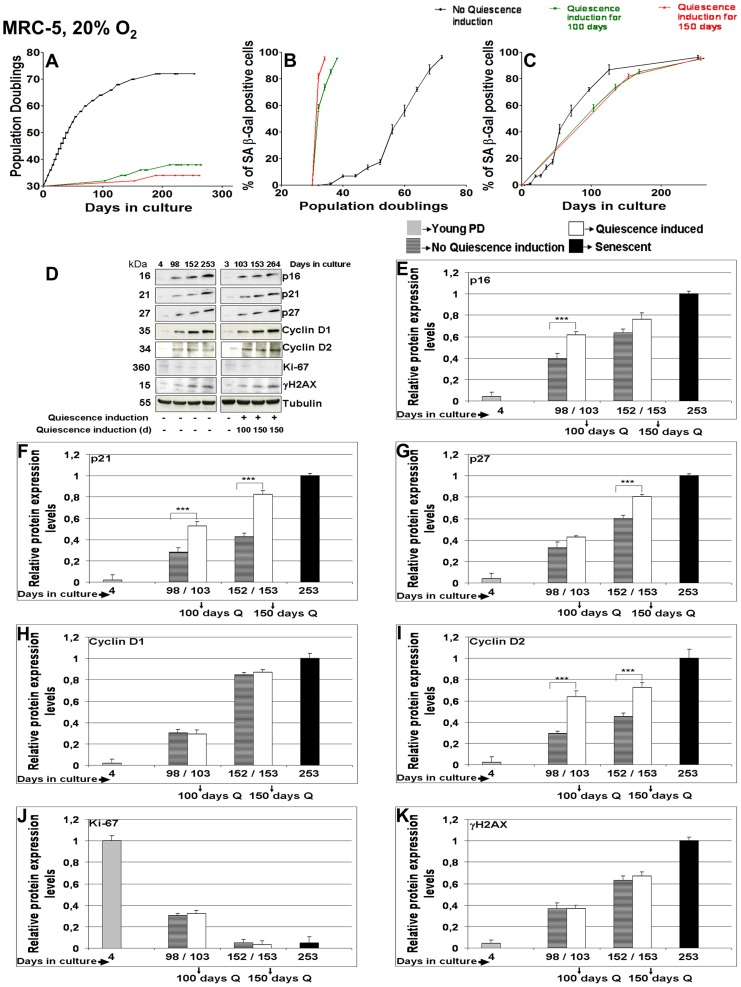
Effect of long term quiescence induction (100 or 150 days) in MRC-5 fibroblasts maintained at 20% O_2_. (**A**) Growth curve of 3 independent MRC-5 fibroblast cell lines (control with no quiescence induction, and cell lines where quiescence was induced for 100 or 150 days respectively by contact inhibition and then maintained in culture till they approached senescence) maintained in culture at 20% O_2_ as triplicates from an early PD until senescence at late PDs. Each growth curve is measured in triplicate. Data points of all measurements are displayed (not the mean). (**B & C**) Percentage of SA-β gal positive cells at different time points of their growth in culture in the control MRC-5 fibroblast cell line and in the cell lines where quiescence was induced for 100 or 150 days respectively. Fig. 2 B and C are plotted with PDs and days in the y-axis respectively. Each curve is measured in triplicate, the mean value is displayed with error bar (± S.E). (**D**) The blots show the protein expression levels of p16, p21, p27, Cyclin D1, Cyclin D2, Ki-67 and γH2A.X in MRC-5 fibroblast cell lines (subjected to different culture conditions of 100 or 150 days quiescence by contact inhibition and no quiescence induction) maintained in culture at 20% O_2_ until they approached senescence at late PD. The up or down-regulation was signified by the presence or absence of the bands in Western Blots. (**E, F, G, H, I, J, K**) Comparison of mean fold change of protein expression levels of p16 (E), p21 (F), p27 (G), Cyclin D1 (H), Cyclin D2 (I), Ki-67 (J) and γH2A.X (K) in MRC-5 cell lines where quiescence was induced for 100 or 150 days by contact inhibition respectively compared to controls at corresponding span of time in culture. Cell lines were maintained at 20% O_2_ as triplicates. The bars indicate the mean ± S.D. *** p<0.001 - significantly different compared to fibroblasts with PD assigned 1. n = 3.

MRC-5 and WI-38 fibroblasts were subjected to long-term quiescence induction by contact inhibition for 100 or 150 consecutive days at 20% O_2_. After release from long-term quiescence, fibroblasts were able to undergo only three (after 100 days of quiescence) or one (after 150 days) further PD(s) (Figs. 2 A and S3). Thus, the replicative life span of long-term quiescence fibroblasts is severely reduced consistent with previous observations in fibroblasts [28] and in yeast [75]. WI-38 fibroblasts released from long term quiescence showed >70% SA-β Gal positive cells after 100 and 150 days of quiescence (S3 Fig.), whereas corresponding MRC-5 cells showed >55% and >80% SA-β Gal positive cells after 100 and 150 days of quiescence induction, respectively (Fig. 2 B and C).

The influence of long-term quiescence on WI-38 and MRC-5 cells was further analyzed by measuring the levels of markers for proliferation and cell cycle arrest. For both cell types, the proliferation marker Ki-67 strongly decreased reaching lowest values in the senescent state (Figs. 2 D, J and S3). The levels of all three cell cycle arrest markers p21, p16, and p27 were increased after release from long-term quiescence (100 and 150 days) compared to controls at corresponding PDs (Figs. 2 D, E, F, G and S3). This behavior of p21 and p16 in quiescent cells is in agreement with published observations [1], [2], [18]. We noticed that, compared to controls, the increase of p21 levels, particularly after 150 days of quiescence, was higher than the increase of p16 and p27 levels (Figs. 2 D, F and S3). After long-term quiescence as well as in normally proliferating control cells, in both fibroblast cell lines Cyclin D1 and D2 increased, as described earlier [76]–[78]. In MRC-5 cells, Cyclin D1 levels were very similar to their control levels (Fig. 2 D and H) while Cyclin D2 showed a strong increase at the end of long-term quiescence (Fig. 2 D and I). In contrast, in WI-38 fibroblasts, Cyclin D1 expression levels strongly increased (S3 Fig.) whereas Cyclin D2 levels were significantly higher only in cells at the end of long-term quiescence after 100 days of quiescence but hardly any increase after 150 days (S3 Fig.). For these markers (p16, p21, p27, Cyclin D1 and D2), highest values were measured for MRC-5 and WI-38 cells when being senescent (Figs. 2 D, E, F, G, H, I, J, K and S3). While both fibroblast cell lines, MRC-5 and WI-38, showed a similar overall behavior and comparable marker increases for p16, p21, p27, we observed a clear difference between these two cell lines for Cyclin D1 (compare Fig. 2 H with S3 Fig.), indicating that both cells lines respond individually different to environmental cues, as proposed before [79].

After long-term quiescence induction, the percentage of SA-β Gal positive cells, when plotted versus time in culture, were found to be similar to controls for WI-38 cells, and only slightly delayed for intermediate time periods for MRC-5 cells (Figs. 2 C and S3). We asked if this similar transition into senescence is driven by a similar level of DNA damage. DNA damage foci can be identified by the marker γH2A.X which is recruited to DNA repair sites [80]–[82]. Consistent with published results [20], [83], [84], in both normally proliferating fibroblast cell lines γH2A.X levels increased with age (Figs. 2 K and S3). Indeed, after long-term quiescence an increase of γH2A.X levels, very similar to controls, was observed in MRC-5 as well as in WI-38 fibroblasts (both 100 and 150 days; Figs. 2 D, K and S3).

High-throughput RNA sequencing of MRC-5 cells at PD 32 showed up-regulation of p16, p27, Cyclin D2, Ki-67 and γH2A.X mRNAs after 150 days compared to 9 days of quiescence (unpublished data), correlating with corresponding protein levels. This suggests that up-regulation of the abundance of these proteins during quiescence is due to transcription. We observed that during long-term quiescence MRC-5 or WI-38 fibroblasts cells do not suffer from telomere shorting (data not shown), in agreement with previous reports [28], [85]. Taken together, our observations show that DNA damage accumulation can occur in non-cycling fibroblasts over time independent of the telomere status.

### Long-term quiescence induction at 3% O_2_

**Figure 3:**
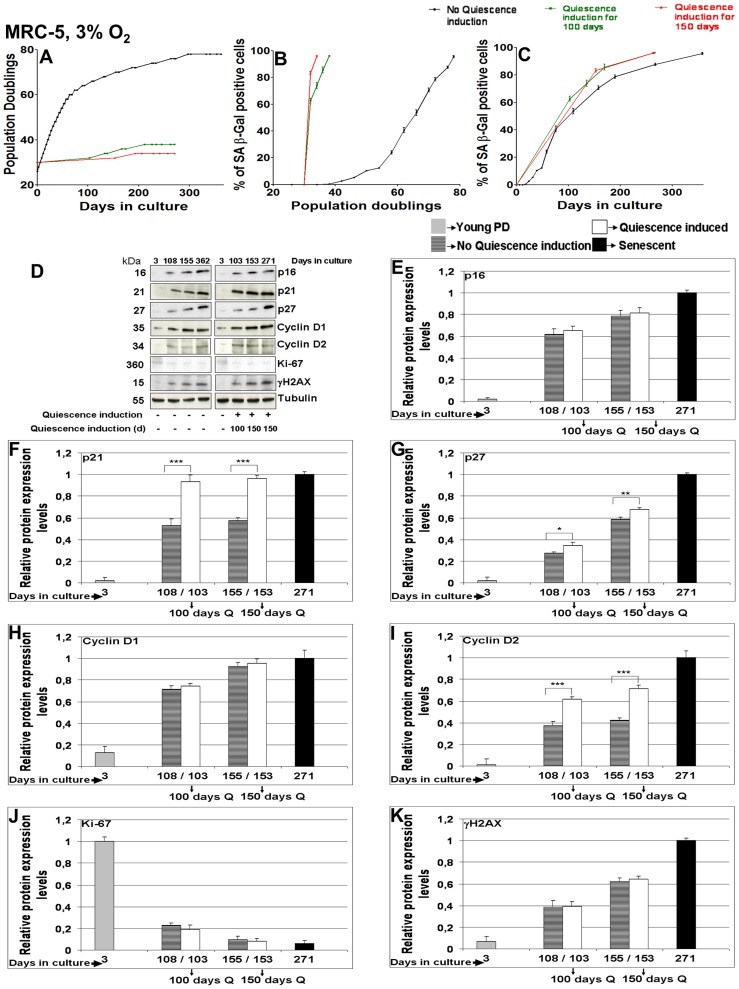
Effect of long term quiescence induction (100 or 150 days) in MRC-5 fibroblasts maintained at 3% O_2_. (**A**) Growth curve of 3 independent MRC-5 fibroblast cell lines (control with no quiescence induction, and cell lines where quiescence was induced for 100 or 150 days respectively by contact inhibition and then maintained in culture till they approached senescence) maintained in culture at 3% O_2_ as triplicates from an early PD until senescence at late PDs. Each growth curve is measured in triplicate. Data points of all measurements are displayed (not the mean). (**B & C**) Percentage of SA-β gal positive cells at different time points of their growth in culture in the control MRC-5 fibroblast cell line and in the cell lines where quiescence was induced for 100 or 150 days respectively. Fig. 3 B and C are plotted with PDs and days in the y-axis respectively. Each curve is measured in triplicate, the mean value is displayed with error bar (± S.E). (**D**) The blots show the protein expression levels of p16, p21, p27, Cyclin D1, Cyclin D2, Ki-67 and γH2A.X in MRC-5 fibroblast cell lines (subjected to different culture conditions of 100 or 150 days quiescence by contact inhibition and no quiescence induction) maintained in culture at 3% O_2_ until they approached senescence at late PD. The up or down-regulation was signified by the presence or absence of the bands in Western Blots. (**E, F, G, H, I, J, K**) Comparison of mean fold change of protein expression levels of p16 (E), p21 (F), p27 (G), Cyclin D1 (H), Cyclin D2 (I), Ki-67 (J) and γH2A.X (K) in MRC-5 cell lines where quiescence was induced for 100 or 150 days by contact inhibition respectively compared to controls at corresponding span of time in culture. Cell lines were maintained at 3% O_2_ as triplicates. The bars indicate the mean ± S.D. * p<0.05, ** p<0.01, *** p<0.001 - significantly different compared to fibroblasts with PD assigned 1. n = 3.

A main source of DNA damage accumulation may be ambient oxygen concentration. We reasoned that reducing O_2_ levels from 20% down to physiological 3% [86] may reduce cellular DNA damage, resulting in lower γH2A.X levels and a delayed transition into senescence [87], [88]. Therefore, MRC-5 and WI-38 were now subjected to long-term quiescence induction by contact inhibition for 100 or 150 consecutive days at 3% O_2_ (Figs. 3 A and S4). After release from long-term quiescence at 3% O_2_, MRC-5 and WI-38 fibroblasts were able to undergo only three or one more PD(s), respectively (Figs. 3 A and S4). We detected >60% and >80% SA-β Gal positive cells in MRC-5 and WI-38 fibroblasts maintained at 3% O_2_ after 100 and 150 days of quiescence, respectively (Figs. 3 B, C and S4). The proliferation marker Ki-67 strongly decreased with time for MRC-5 and WI-38 cells being released from long-term quiescence as well as for proliferating control cells (Figs. 3 D, J and S4). In MRC-5 fibroblasts maintained at 3% O_2_, p16 (Fig. 3 D and E) and Cyclin D1 (Fig. 3 D and H) values were similar to control values and p27 (Fig. 3 D and G) values only slightly increased for cells released from long-term quiescence. In contrast, p21 (Fig. 3 D and F) and Cyclin D2 (Fig. 3 D and I) values strongly increased in cells released from long-term quiescence compared to proliferating control cells. In WI-38 fibroblasts maintained at 3% O_2_, the expression levels of all cell cycle markers analyzed (p16, p21, p27, Cyclin D1 and D2) increased after release from 100 or 150 days of quiescence compared to proliferating controls of corresponding PDs (S4 Fig.). Furthermore, the levels of DNA damage marker γH2A.X revealed no differences between MRC-5 and WI-38 fibroblasts cells after release from long-term quiescence (both 100 and 150 days) compared to normally proliferating controls (Figs. 3 D, K and S4). Importantly, after about 100 or 150 days in culture, γH2A.X levels were very similar in MRC-5 cells either kept under 20% or 3% O_2_ (Figs. 3 K and S4). We therefore conclude that a non-physiological increased oxygen level, at least the one applied here, seems not to be the direct linear source of DNA damage accumulation during quiescence or senescence. The cellular effect of oxygen may be complex: oxygen levels were found to influence the transition into senescence in a non-proportional way with low oxygen having a protective effect only at the end of the cellular lifespan [89], [90].

**Figure 4:**
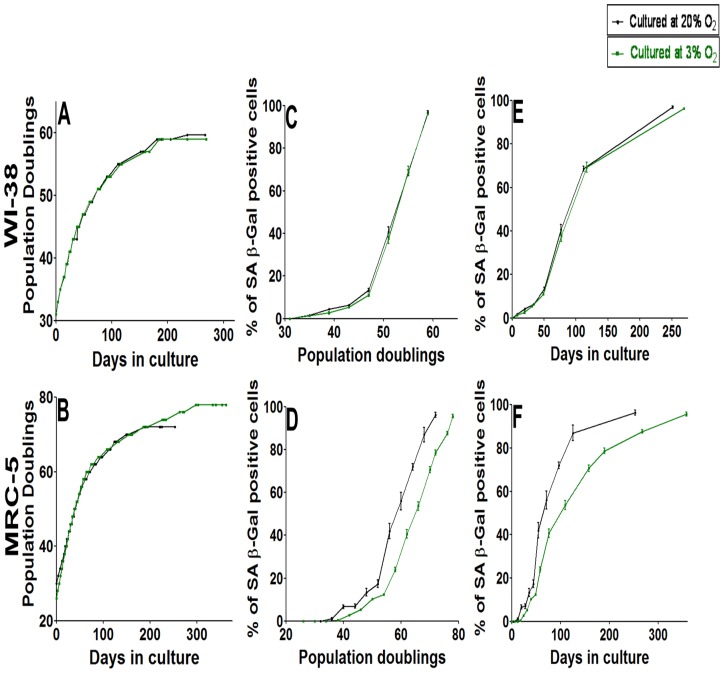
Comparison of the effect of O_2_ levels (3 or 20%) in culture on the growth curve and induction of senescence revealed by β gal in MRC-5 and WI-38 fibroblasts. (**A & B**) Growth curve of 2 independent WI-38 (A) and MRC-5 (B) fibroblast cell lines maintained in culture at 20% or 3% O_2_ till they achieved senescence at late PD. Data points of all measurements are displayed (not the mean). (**C & D**) Percentage of SA-β gal positive cells at different time points of their growth in culture in WI-38 (C) and MRC-5 (D) fibroblast cell lines maintained at 20% or 3% O_2_. The figures were plotted with PDs on the x-axis (**E & F**) Percentage of SA-β gal positive cells at different time points of their growth in culture in WI-38 (E) and MRC-5 (F) fibroblast cell lines maintained at 20% or 3% O_2_. The figures were plotted with days in culture on the x-axis. Each curve is measured in triplicate, the mean value is displayed with error bar (± S.E). n = 3.

We then asked if the low oxygen level had an influence on the aging of these fibroblast cells. We found that WI-38 fibroblasts, maintained at 3% O_2_, approached senescence quantitatively in the same way as those maintained at 20% O_2_, as shown by the growth curve (Fig. 4 A) and the percentage of SA-β Gal positive cells (Fig. 4 C and E). Instead, MRC-5 fibroblasts, maintained at 3% O_2,_ had a longer lifespan compared to cells kept at 20% O_2_ (Fig. 4 B). Under low O_2_ levels, MRC-5 cells were able to divide even after 300 days in culture while cells under 20% O_2_ underwent their last division after ca. 185 days, and MRC-5 cells at 3% O_2_ were able to undergo considerably more PDs (cumulative PD = 78) compared to those at 20% O_2_ (cumulative PD = 72). Parallel to the MRC-5 growth curve (Fig. 4 B), the transition into senescence is delayed for MRC-5 maintained at 3% O_2_ (Fig. 4 D and F). The results indicate a clear difference in the behavior of WI-38 compared to MRC-5 cells: reducing the oxygen level from 20% to 3% did not change the aging of WI-38 cells but delayed aging in MRC-5 cells. Considerable cell-strain variability in response to oxygen had been observed before [87].

### HES1 is up-regulated in long-term quiescent and senescent fibroblast cells

**Figure 5:**
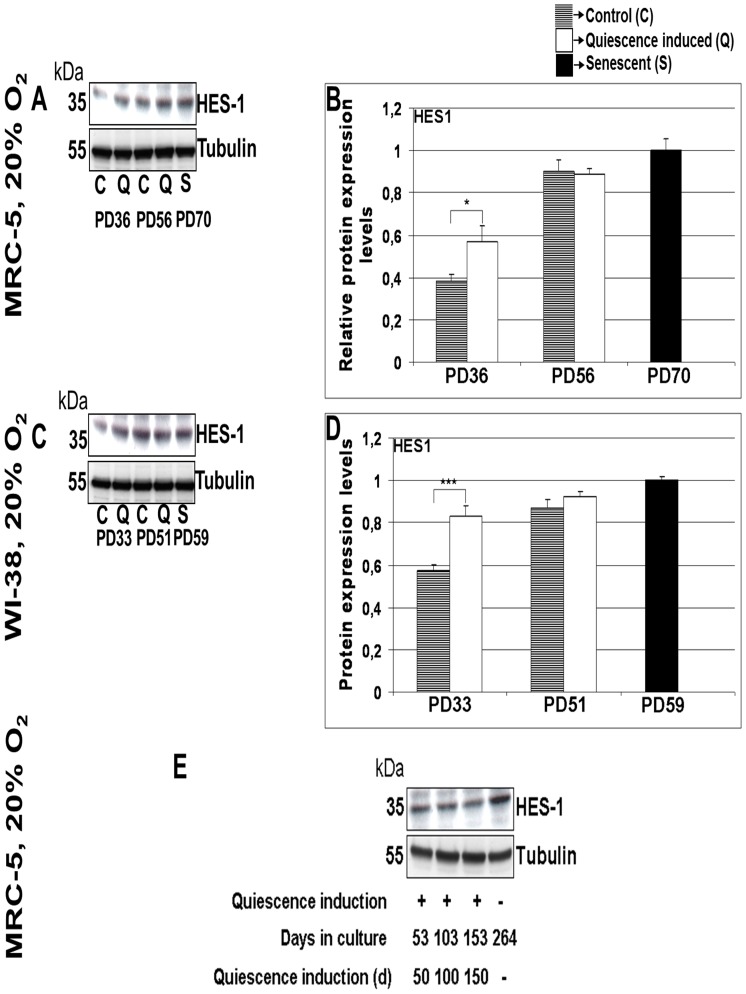
Expression levels of HES1 in MRC-5 and WI-38 fibroblasts subjected to short term or long term quiescence induction. (**A**) The blot show the protein expression levels of HES1 in two MRC-5 fibroblast cell lines (control with no quiescence induction and a cell line where quiescence was induced 3 times separately for a span of 9 days) maintained at 20% O_2_ at different stages of their span in culture. The up or down-regulation was signified by the presence or absence of the bands in Western Blots. (**B**) Comparison of mean fold change of protein expression levels of HES1 in 3 times quiescence induced MRC-5 cell lines and control MRC-5 cell lines maintained in culture as triplicates. (**C**) The protein expression levels of HES1 in two WI-38 fibroblast cell lines (control with no quiescence induction and a cell line where quiescence was induced 3 times separately for a span of 9 days) maintained at 20% O_2_ at different stages of their span in culture. (**D**) Comparison of mean fold change of protein expression levels of HES1 in 3 times quiescence induced WI-38 cell lines and control WI-38 cell lines maintained in culture as triplicates. The bars indicate the mean ± S.D. * p<0.05, *** p<0.001 - significantly different compared to fibroblasts with PD assigned 1. n = 3 (**E**) The protein expression levels of HES1 in MRC-5 fibroblast cell lines maintained at 20% subjected to 50, 100 or 150 days of quiescence by contact inhibition and in senescent state. The up or down-regulation was signified by the presence or absence of the bands in Western Blots. n = 2.

In quiescent cells, the transcriptional repressor HES1 is up-regulated and, during a period of several days, prevents transition into senescence although during quiescence p21 is up-regulated [27]. Thus, HES1 guarantees reversibility of quiescent cells into the cell cycle. Consistent with these observations, we detected only minor influences of short term quiescence on the number of SA-β Gal positive cells compared to normally proliferating control cells (Fig. 1) despite detecting p21 up-regulation (S1 and S2 Figs.). After short-term (9 days) quiescence of MRC-5 and WI-38 cells, we observed an up-regulation of HES1 compared to the HES1 level in normally proliferating cells (Fig. 5 A, B, C and D) while after 3 times 9 days of quiescence, the HES1 levels had further increased and now are similar to the HES1 levels in control cells (Fig. 5 A, B, C and D). We then asked if HES1 remains up-regulated also after long-term quiescence. We found high levels of HES1 in MRC-5 (Fig. 5 E) as well as WI-38 cells after 100 and 150 days of quiescence independent of 3% or 20% oxygen levels. Surprisingly, we find high levels of HES1 in both cells types also when being senescent (Fig. 5). In order to check that the antibody indeed recognizes HES1, we cloned HES1 in fusion with GFP and verified that the HES1 antibody recognizes the fusion protein which in parallel is identified by an anti-GFP antibody (data not shown). Thus, HES1 remains up-regulated; however, after long-term quiescence it is no longer able to prevent transition into senescence (as indicated by our data above).

### Quiescent as well as senescent fibroblast cells are protected from transition into apoptosis

**Figure 6:**
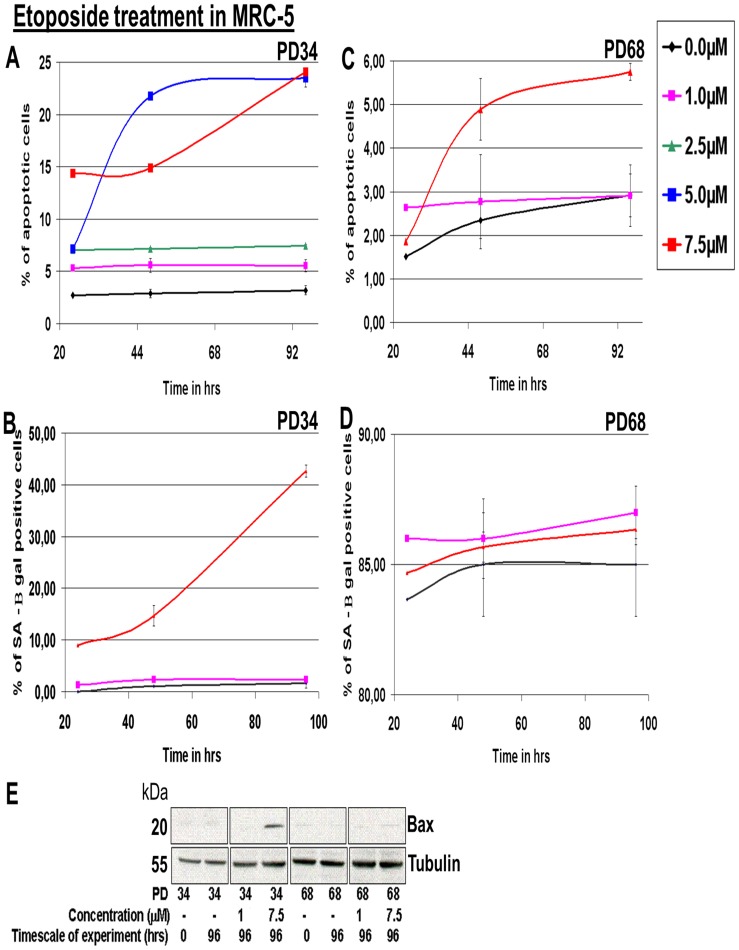
Impact of Etoposide treatment in young and old PD MRC-5 fibroblasts. (**A**) Percentage of apoptotic cells in MRC-5 fibroblast cell lines (young PD = 34) treated with different concentrations of Etoposide for different time spans (**B**) Percentage of SA-β gal positive cells in MRC-5 fibroblast cell lines (young PD = 34) treated with different concentrations of Etoposide for different time spans (**C**) Percentage of apoptotic cells in MRC-5 fibroblast cell lines (old PD = 68) treated with different concentrations of Etoposide for different time spans (**D**) Percentage of SA-β gal positive cells in MRC-5 fibroblast cell lines (old PD = 68) treated with different concentrations of Etoposide for different time spans. In each instance, MRC-5 fibroblasts were maintained in culture at 20% O_2_ as triplicates. In A & C, the bars indicate the mean ± S.D. In B & D error bars indicate ± S.E). (**E**) The blots show absence or induction of apoptotic protein Bax in MRC-5 fibroblast cell lines (young PD = 34 & old PD = 68) treated with 1.0 or 7.5 µM of Etoposide for 96 hrs compared to controls (0 hrs). The MRC-5 fibroblasts were maintained in culture at 20% O_2_. The up or down-regulation was signified by the presence or absence of the bands in Western Blots. n = 3.

To avoid danger to the cell and its framing tissue, not only normally proliferating but also quiescent cells must be able to react to harmful stress by inducing apoptosis. We therefore analyzed the potential of fibroblast cells for apoptosis induction. As inducing agents, we applied Etoposide [91] as well as Staurosporine [92] and detected the apoptotic state by immunostaining of Bax phosphorylation [93], [94]. Young MRC-5 fibroblasts at PD 34 were exposed to increasing concentrations of Etoposide (0.0 to 7.5 µM). As detected by immunostaining in Western blots, 5.0 µM or 7.5 µM of Etoposide resulted in 23% apoptotic MRC-5 fibroblasts after 96 hrs of treatment in culture compared to 3% apoptotic control cells at 0.0 µM Etoposide (Fig. 6 A). Treatment of young MRC-5 fibroblasts with 7.5 µM Etoposide also resulted in the induction of pro-apoptotic Bax protein (Fig. 6 E). Exposure to 7.5 µM Etoposide for 96 hrs revealed an induction of ca. 43% SA-β Gal positive cells while lower Etoposide concentrations did not induce senescence (Fig. 6 B). In contrast, when old MRC-5 fibroblasts at PD 68 were exposed to Etoposide for 96 hrs, 2–3% induction of apoptosis was observed for 1 µM and only a slight increase from 3–6% at 7.5 µM (Fig. 6 C). This minor induction could be explained by the small amount of non-senescent cells in the population being induced to apoptosis. Consistent with these results, Bax proteins were not induced as revealed by Western blots (Fig. 6 E). Treatment of old MRC-5 fibroblasts at PD 68 displayed about 85% SA-β Gal positive cells independent of Etoposide concentrations (0.0, 1.0 or 7.5 µM; Fig. 6 D). A similar behaviour was observed when the cells were treated with Staurosporine: increasing concentrations of 0.0, 0.1, 0.5, 1.0, and 2.0 µM Staurosporine in the serum of young MRC-5 fibroblasts at PD 34 resulted in an increase in the percentage of apoptotic cells. 1.0 and 2.0 µM Staurosporine were able to induce apoptosis in 20–25% of cells. The presence of apoptotic cells was also detected by Bax protein induction. 2.0 µM Staurosporine induced senescence in more than 50% of the cells. When old MRC-5 fibroblasts (PD 68) were exposed to 2.0 µM Staurosporine for 96 hrs, an only minor induction of apoptosis was detected (from 2% to 4%) while about 85% of the cells were SA-β Gal positive (Fig. S5). In summary, apoptosis can be induced in young fibroblast cells; however, even at high concentrations of inducing agents, apoptosis is induced only to a minor extent in a senescent fibroblast cell population, consistent with [95].

Then we asked if high concentrations of these agents can induce apoptosis in quiescent cells. Treating short-term (9 day) quiescent MRC-5 fibroblasts (PD 35) for 96 hours with 7.5 µM Etoposide or 2.0 µM Staurosporine revealed 16% and 13% apoptotic cells, respectively (S6 Fig.), as indicated by Bax phosphorylation. Thus, apoptosis can be induced in short-term quiescent cells, however, to a reduced amount compared to normally proliferating cells. In long-term (150 days) quiescent MRC-5 fibroblasts (PD 32), 7.5 µM Etoposide or 2.0 µM Staurosporine were unable to induce significant levels of apoptosis (S6 Fig.): the percentage of apoptotic cells of 4–5% in young (PD 32) fibroblasts subjected to 150 days of quiescence were similar to the percentage of apoptotic cells observed in senescent fibroblasts (PD 68) (S6 Fig.). Supporting this result, the treatment of long-term quiescence fibroblasts (150 days) with either Etoposide or Staurosporine did not induce the pro-apoptotic protein Bax (S6 Fig.). Thus, consistent with earlier findings [96], during quiescence the cells lose their potential for apoptosis induction, as do normally proliferating cells during their life span.

### Differential regulation of mRNA expression in long-term quiescent fibroblasts is similar to that in senescent cells

We observed that long-term quiescent fibroblasts transit into senescence by a similar rate as do normally proliferating cells (Figs. 3 C and S4). We speculated that this similarity in rates may be due to similar cellular mechanisms ruling this transition in both cellular states. We therefore compared gene expression profiles, measured by RNA-seq, of young MRC-5 cells (PD 32) with long-term quiescent MRC-5 fibroblasts (subjected to 150 days contact inhibition). We identified 1,499 up- and 1,216 down-regulated differentially expressed genes (DEGs). As a next step, we intersected these DEGs with senescence-regulated genes identified by [97] which resulted in 60 up- and 194 down-regulated DEGs (S7 Fig.). Identification of the common significantly enriched GO terms and KEGG pathways between the two data sets (150 days quiescent versus senescent fibroblasts) revealed that “cell cycle (GO:0007049)”, “ATP binding (GO:0005524)”, and “DNA repair (GO:0006281)” were among the most significantly down-regulated pathways (data not shown). A number of similarly regulated KEGG pathways found by gene set enrichment analysis, were obtained for both datasets (S8 Fig.), again identifying “repair” as one of the commonly down-regulated pathways. Thus, both, quiescent and normally proliferating cells, transit into senescence by modifying the same pathways (in particular down-regulating the DNA repair pathway) by similar amounts, i.e. they transit into senescence due to similar cellular processes.

## Discussion

Several independent signals (like mitogen withdrawal, contact inhibition, loss of adhesion, or RAS induction) induce cellular quiescence, a reversible cell cycle arrest, by up-regulation of the CDK inhibitor p21. Each of these signals causes regulation of a unique set of genes known to terminate growth and division [15]. Nevertheless, underlying this diversity of states, the expression of a set of genes was found to be independent of the quiescence induction signal, and thus describing a general quiescent state, regardless of the signal that induced it [15]. Reversibility of quiescence into the cell cycle is insured by suppressing the transition into terminal differentiation [15]. Furthermore, short-term quiescent cells are described to be protected against transition into senescence by up-regulation of the transcriptional repressor HES1 [27]. Considered to being permanently reversible, long-term quiescent cells would be cancer-prone: although not dividing, the quiescent cells accumulate DNA damage (as shown here), due to their re-directed but still high metabolic activity [9]. Low level DNA damage is considered to be routinely repaired (maintenance, see [79]) while, in normally proliferating cells, harmful damage induces apoptosis. However, after long-term quiescence, as we show here in agreement with others [96] apoptosis induction is strongly suppressed which, after return into the cell cycle, immediately increases the potential for tumor development. In order to avoid this dangerous fate, while being quiescent for longer periods of time, cells might transit into senescence. Indeed, quiescent HFF fibroblast cells were found to become senescent [28]. This however, when being of general nature, would contradict the HES1 protection mechanism and the definition of quiescence, i.e. that all quiescent cells can return into the cell cycle.

We resolved this contradiction in cultured cells by analyzing further human primary fibroblasts (to what extent these results hold *in vivo*, remains to be shown). We induced short-term and long-term quiescence in MRC-5 and WI-38 fibroblasts. After short-term (9 day) quiescence we observed p21 and p27 up-regulation but no induction of p16 (S1 and S2 Figs.), in agreement with earlier findings [1], [18]. In these cells, HES1 was up-regulated (Fig. 5 A, B, C, D); DNA damage was not induced as indicated by low γH2A.X levels (S1 and S2 Figs.), and the growth curve was similar to that of normally proliferating control cells. These and the SA-β Gal results show that, after short-term quiescence, these cells were in a reversible cell cycle arrested state with no indications for transition into senescence. This marker pattern strongly changed after long-term quiescence. Still, after 100 and 150 days of quiescence, p21 and p27 were up-regulated, but now also p16, while the proliferation marker Ki-67 decreased, the DNA damage marker γH2A.X increased, and the growth curve strongly changed (Fig. 2). The number of SA-β Gal positive cells, when plotted versus population doublings (PDs), showed a markedly different behavior compared to that of normally proliferating control cells. We observed that during long-term quiescence, DNA damage accumulates at comparable amounts as in control cells (as detected by γH2A.X levels), and most of the quiescent cells transit into senescence (as indicated by a similar increase of SA-β Gal positive cells), with only a small part of the population still being proliferative active (Ki-67 positive).

Thus, not all quiescent cells are able to return into the cell cycle but, after release from quiescence, the cell population is re-established out of the small non-senescent cell fraction. When indeed during long-term quiescence the proliferative cell fraction of the population decreases, then, after release from quiescence, re-establishing a proliferative population must take the longer, the longer the cells have been quiescent. This prediction agrees with our observations and published findings: cells deprived of serum for weeks rather than days, took longer to re-enter the cell cycle [98]–[100], correlating with cell size [8]. We observed that cells having experienced quiescence subsequently grow slower (Fig. 1 G and H). Furthermore, the time required to grow to confluency (time required for 1 PD) is clearly longer after 100 or 150 days of quiescence than required by young proliferating cells, instead it relates to the time required by old cell populations close to senescence.

When plotting the number of SA-β Gal positive cells versus number of days in culture, during and after long-term quiescence the cells behaved rather similar to control cells. This similar transition rate into senescence made us speculate that with time (days in culture) during quiescence as well as during normal proliferation, similar cellular processes within these cells might rule this transition. We therefore compared the gene expression profile of young with that of long-term quiescent fibroblasts, and determined which genes are either up- or down-regulated when cells are in long-term quiescence. Interestingly, we found DNA repair pathway genes down-regulated, explaining accumulation of DNA damage in long-term quiescent cells. These differentially expressed genes were compared to those recently found when comparing young and senescent fibroblast cells [97]. In both data sets, we identified a number of pathways which are differentially regulated in a similar way. For example [97] found that also in senescent cells, the DNA repair pathway is down-regulated. Thus, quiescent and normally proliferating cells transit into senescence not only in similar time frames but also due to similar cellular mechanisms. As observed here for fibroblasts, during quiescence also hematopoietic stem cells (HSCs) were recently found to accumulate DNA strand breaks [101], and also these cells transcriptionally attenuated the DNA damage response and repair (DDR) pathway.

Our observations suggest the following model for fibroblasts in cell culture. Cells proliferate with the rate “r” [79]. Whenever repair is required, the cells exit the cell cycle for a short period of time by up-regulating the CDK inhibitor p21, carry out the repair and return into the cell cycle. This “maintenance” reduces the rate of cell growth [79]. When cells are unable to complete the repair process, the cells transit into senescence, with only a very minor part of the population still being proliferatively active (as indicated by the low but detectable Ki-67 levels). This transition correlates with the up-regulation of the CDK inhibitor p16. The p53-p21 pathway is connected to the p16-pRB pathway [55], [59], with p16 being up-regulated after some time delay (estimated from our data: ca. 30–40 days). We suggest that this delayed response of the p16 pathway is the time frame the cells allow for cell cycle reversible DNA damage response, the process of normal cellular maintenance. Once this time is used up, p16 up-regulation induces a permanent cell cycle arrest, which in human cells is not necessarily irreversible [57]. Consistent with this view, (i) murine p16 knock-out cells do not become senescent [102], [103], (ii) p16 is inactivated in many human tumors [104], [105], and (iii) ectopic expression of p16 in human cancer cell lines being negative in p16 expression, induced growth arrest and senescence [106], [107]. Here we show that a corresponding process also takes place during long-term quiescence; indeed, quiescent and cells in maintenance transit into senescence due to similar cellular mechanisms. Although we detected quantitative differences between cell types, the same qualitative behavior was observed between MRC-5 and WI-38 cells. It remains to be shown if this model also holds true for cells in tissue. We observed that apoptosis could be induced in young but hardly in senescent cells [91], [95] in contrast to observations in HS74 [108] and PAEC cells [109], discussed in [37]. Correspondingly, after short-term (9 days) but hardly after long-term (150 days) quiescence, apoptosis could be induced, again indicating a complementary development over time in proliferating and quiescent cells. Interestingly, senescent cells cannot transit into quiescence [100].

Fibroblast cells can divide up to about 70 times before stably arresting and becoming senescent [29]. Senescent cells, being arrested in the cell cycle, remain metabolically active but prevent cancer development. Shortening of telomeres is considered to be an origin for this transition into senescence: telomere shortening might act as a counting mechanism (“replicometer”) triggering replicative senescence in normal diploid cells since these cells do not contain telomerase [110], [111]. Telomere shortening triggers a p53/p21-dependent cell cycle arrest through accumulation of G-rich single stranded DNA fragments. This prevents replication of damaged DNA, allowing sufficient time for repair [112], [113]. If cells are unable to repair the DNA damage, p16 is up-regulated and the arrest becomes permanent [57]. If telomere shortening would be the main mechanism triggering senescence, during non-replicating quiescence, cells should not transit into senescence and time in the quiescent state would not count for their replicative life span since telomeric attrition strongly depends on cell division, both *in vitro* [85], [114] and *in vivo* [114]. In contrast to this and consistent with [28], we find that quiescent human fibroblast cells age with a rate similar to normally proliferating cells. Thus, in human fibroblasts, telomere shortening cannot be the dominant mechanism triggering senescence but factors other than mere telomere length, or different mechanisms must drive this transition [67]. Consistently, in human cells expression of telomerase did not reverse the senescence arrest [57]. Further arguments support this notion: individual cells from clonally derived populations show heterogeneous division potential [115], and the fraction of senescent cells, present in a large population, increases progressively with PDs, and not most of them together at a given PD. This indicates that the lifespan of an individual cell lineage is not controlled simply by telomere shortening during each round of cell division, but instead also by sensing genotoxic stresses [116] or other independent mechanisms upstream of telomere shortening [37], like for example: enhanced cellular ability to bind DNA-ends may be important for longevity [66], and telomerase might down-regulate the p16 pathway [103], [117].

We speculated that high oxygen levels might induce the increasing amount of DNA damage. We reduced the extracellular oxygen levels from 20% to physiological 3% and detected no or only minor quantitative differences. Thus, the increasing amounts of DNA damage and the increase of SA-β Gal positive cells with time in culture are not mainly due to high (20%) oxygen levels; instead we consider internal cellular processes like metabolic effects responsible for this. Furthermore, when high oxygen stress (inducing DNA damage and not telomere shortening) would trigger senescence in quiescent cells, lower oxygen concentrations should delay the transition into senescence [87], [88]. However, in quiescent WI-38 cells the transition into senescence was not altered when reducing the oxygen level from 20% to 3%; thus, WI-38 cells do not respond to this difference in stress. MRC-5 did sense the reduction in stress level: during quiescence, the increase of the number of SA-β Gal positive MRC-5 cells was slightly delayed. Nevertheless, MRC-5 cells became senescent also under low oxygen levels. These results clearly identify quantitative differences in the behavior of these human cell lines, as detected before [79], [87]. Thus, other cellular processes must be involved in senescence induction [49], [118] which must be active in non-replicating quiescent cells also at low oxygen levels [119]. We speculate that cellular maintenance is the common basic mechanism driving normally proliferating as well as quiescent cells into senescence.

## Materials and Methods

### Cell culture

Primary human lung fibroblasts MRC-5 (derived from normal lung tissue of a 14 week old male fetus) and WI-38 (derived from 12 week old female fetus) were obtained from ATCC (LGC Promochem GmbH, Wesel, Germany) at young population doublings (PD) of 26-28. Cells were cultured in Dulbeccos modified Eagles medium (DMEM) with L-Glutamine, low glucose (PAA Laboratories, Pasching, Austria), supplemented with 10% fetal bovine serum (FBS) (PAA Laboratories, Pasching, Austria). Cell culture was carried out under normal air conditions in a 9.5% CO_2_ atmosphere at 37°C. For subculturing procedures, 1x PBS (pH 7.4) (PAA Laboratories, Pasching, Austria) and trypsine/EDTA (PAA Laboratories, Pasching, Austria) were used. Fibroblast cultures approaching confluency were splitted at a ratio of 1∶4. For replicative senescence, fibroblasts were maintained until the end of their lifespan (>95% of cells SA-β Gal positive). Quiescence induction was performed by contact inhibition or serum starvation. For contact inhibition, quiescence was induced either repetitively (3 times for 9 days during the culture span of the fibroblasts) or in long-term (100 or 150 days of consecutive quiescence induction). In MRC-5 fibroblasts, short-term quiescence was induced during PDs 36, 44 and 56 whereas in WI-38 fibroblasts, short-term quiescence was induced at PDs 33, 43 and 51. Once the cells were confluent at their respective PDs, they were left in the confluent state for 9 days with media change (containing 10% FBS) every 3 days (contact inhibition). Then the cells were split and transferred back to normal culture conditions with subsequent canonical splitting. With respect to the long-term quiescence induction, the cells were allowed to reach confluence at early PDs and were left in a confluent state for 100 or 150 days. In this instance, media (containing 10% FBS) was changed every 3 days. Otherwise, quiescence was induced for 8 days by incubation of cells with serum-deprived medium, DMEM supplemented with 0.5% FBS (serum starvation). After addition of normal growth medium, cells resumed proliferation.

### SA-β Gal activity assay

SA-β Gal activity was determined as described by [38]. SA-β Gal was measured for fibroblasts in culture at every four PDs, analyzing mean values ± standard deviation of 3×60 cells, each.

### Western blotting

For Western blotting, 10^4^ cells/µl were used per lane. Immunodetection was performed using 5%-powdered milk in PBS-T (1xPBS, pH 7.4 and 1% Tween20) for blocking (Roth, Germany). Primary antibodies, anti-p21 mouse antibody (OP64; Calbiochem; dilution 1∶200), anti-p16 mouse antibody (550834; BD Pharmingen; 1∶200), anti-p27 rabbit antibody (sc-528; Santa Cruz; 1∶200), anti-γH2AX (07-164; Millipore; 1∶50), anti-Cyclin D1 rabbit antibody (ab16663; Abcam; 1∶500), anti-Cyclin D2 mouse antibody (ab3805; Abcam; 1∶500), anti-Ki-67 mouse antibody (ab6526; Abcam; 1∶200), anti-HES1 rabbit antibody (sc-25392; Santa Cruz; 1∶200), anti-Bcl-2 (IMG-80093; IMGENEX; 1∶200), anti-Bax (IMG-80165; 1∶250) and anti-tubulin mouse antibody (T-9026; SIGMA-Aldrich; 1∶5000) were diluted as indicated in 5%-powdered milk (in PBS-T) and incubated for one hour at room temperature. Washing steps were performed 3×10 min in 1×PBS-T. The secondary horseradish peroxidase-labeled antibodies (Jackson Immuno Research Lab) were incubated for 1 hr at room temperature. Detection of horseradish peroxidase was performed using ECL-detection system and radiographic film (GE Healthcare, Germany). After film development, quantification of signal intensities of the bands in the Western blots was carried out using Metamorph software.

### Treatment of human embryonic lung fibroblasts with apoptotic agents

Two well established apoptotic inducers; Staurosporine [92], [120]–[122] and Etoposide [91], [123] were used in this study. Young (PD = 34) and aged (PD = 68) MRC-5 fibroblasts were treated with different concentrations of Staurosporine (0.1, 0.5, 1.0, 2.0 µM) or Etoposide (1.0, 2.5, 5.0, 7.5 µM) for different time spans (24, 48, 96 hrs) and maintained in culture at 20% O_2_. The fibroblasts were subjected to Etoposide or Staurosporine treatment after every 24 hrs of their span in culture. The percentage of SA-β Gal positive and apoptotic cells were investigated in the MRC-5 fibroblasts after different spans of treatment with Staurosporine and Etoposide.

### Detection of apoptotic cells by Hoechst staining using flow cytometry

A BD FACS Canto II was used for flow cytometry. Ca. 10^6^/ml of MRC-5 fibroblasts cells treated with different concentrations of apoptotic agents were stained with Hoechst 33342 and propidium iodide staining to detect the percentage of apoptotic cells [120]. Hoechst 33342 blue fluorescence dye (excited by 405 nm laser line, emission detected using a 450±50 nm band pass filter) stains the condensed chromatin of apoptotic cells brighter than the chromatin of non-apoptotic cells, while the red fluorescence dye propidium iodide (excited by 488 nm laser line, emission detected using a 670 nm long pass filter) permeates only into dead cells, enabling the differentiation of dead from apoptotic cells. The percentage of apoptotic cells was then retrieved by performing flow cytometry [124]. Experiments were performed according to the protocols of the manufacturer (BD Bioscience) and fluorescence signals were analyzed using the FACS Diva software 6.1.7 (BD Bioscience).

### High-throughput RNA sequencing

For quality check, total RNA was analyzed using Agilent Bioanalyzer 2100 (Agilent Technologies) and RNA 6000 Nano Kit (Agilent) to ensure appropriate RNA quality in terms of degradation. Total RNA was used for Illumina library preparation and next-generation sequencing. Around 2.5 µg total RNA was used for indexed library preparation using Illumina's TruSeq RNA Sample Prep Kit v2 following the manufacturer's instruction. Libraries were pooled and sequenced using Illumina HiSeq2000 and HiSeq2500 sequencing machines in single read mode with 50 cycles using sequencing chemistry v3. Reads were extracted in FastQ format using CASAVA v1.8.2 (Illumina).

### RNA-seq data analysis

Raw data sequencing results were received in FastQ format. Read mapping was performed using Tophat 2.0.6 [125] and the human genome references assembly GRCh37.66 obtained from Ensembl. Uniquely mapped reads were counted for all genes using featureCounts [126]. RPKM values were computed using exon lengths provided by featureCounts and the sum of all mapped reads per sample. Differentially expressed genes (DEGs) were identified using the DESeq [127], edgeR [128] and baySeq [129] statistical software analysis tools. The resulting p-values were adjusted using the Benjamini and Hochberg's approach for controlling the false discovery rate (FDR) [130]. For comparing DEGs from this study with data by [97] the same statistical cutoffs have been used (FDR of all three tests <0.05 and absolute log2 fold-change >1). P-values for the overlap of gene lists were calculated using the web tool at http://nemates.org/MA/progs/overlap_stats.html with a total number of genes of 16,035 representing the number of comparable genes between both datasets. Singular enrichment analysis was performed for the intersection of either up- and down-regulated DEGs found in both studies using DAVID [131]. Generally applicable gene set enrichment for pathway analysis (GAGE) [132] was used in order to detect significantly regulated KEGG pathways (FDR adjusted p<0.01) while log2 fold-changes and normalized gene counts have been used for data by [97] and data of this study, respectively.

### Statistical analysis of the samples

Where appropriate, quantitative results were examined for statistical significance using paired two-sample type 2 Student's t-tests assuming equal variances; p values are presented where appropriate.

### Ethics Statement

The human fibroblast cell lines (MRC-5 and WI-38) used in this investigation was ordered from ATCC. A number of senescence related studies has been undertaken using MRC-5 and WI-38 [133], [134], [135] fibroblast cell lines.

## Supporting Information

S1 Fig **Effect of short term quiescence induction on protein expression levels of a number of cell cycle associated genes and a marker for DNA damage in MRC-5 fibroblasts.** (**A**) The blots show the protein expression levels of p16, p21, p27, Cyclin D1, Cyclin D2 and γH2A.X in two MRC-5 fibroblast cell lines (control with no quiescence induction and a cell line where quiescence was induced 3 times separately for a span of 9 days) maintained at 20% O_2_ at different stages of their span in culture. The up or down-regulation was signified by the presence or absence of the bands in Western Blots. (**B, C, D, E, F, G**) Comparison of mean fold change of protein expression levels of p16 (B), p21 (C), p27 (D), Cyclin D1 (E), Cyclin D2 (F) and γH2A.X (G) in 3 times quiescence induced MRC-5 cell lines and control MRC-5 cell lines maintained in culture as triplicates. The bars indicate the mean ± S.D. ** p<0.01, *** p<0.001 - significantly different compared to fibroblasts with PD assigned 1. n = 3.
(TIF)

S2 Fig **Effect of short term quiescence induction on protein expression levels of a number of cell cycle associated genes and a marker for DNA damage in WI-38 fibroblasts.** (**A**) The blots show the protein expression levels of p16, p21, p27, Cyclin D1, Cyclin D2 and γH2A.X in two WI-38 fibroblast cell lines (control with no quiescence induction and a cell line where quiescence was induced 3 times separately for a span of 9 days) maintained at 20% O_2_ at different stages of their span in culture. The up or down-regulation was signified by the presence or absence of the bands in Western Blots. (**B, C, D, E, F, G**) Comparison of mean fold change of protein expression levels of p16 (B), p21 (C), p27 (D), Cyclin D1 (E), Cyclin D2 (F) and γH2A.X (G) in 3 times quiescence induced WI-38 cell lines and control WI-38 cell lines maintained in culture as triplicates. The bars indicate the mean ± S.D. ** p<0.01, *** p<0.001 - significantly different compared to fibroblasts with PD assigned 1. n = 3.
(TIF)

S3 Fig **Effect of long term quiescence induction (100 or 150 days) in WI-38 fibroblasts maintained at 20% O_2_.** (**A**) Growth curve of 3 independent WI-38 fibroblast cell lines (control with no quiescence induction, and cell lines where quiescence was induced for 100 or 150 days respectively by contact inhibition and then maintained in culture till they approached senescence) maintained in culture at 20% O_2_ as triplicates from an early PD until senescence at late PDs. Each growth curve is measured in triplicate. Data points of all measurements are displayed (not the mean). (**B & C**) Percentage of SA-β gal positive cells at different time points of their growth in culture in the control WI-38 fibroblast cell line and in the cell lines where quiescence was induced for 100 or 150 days respectively. S3B and S3C Figs. are plotted with PDs and days in the y-axis respectively. Each curve is measured in triplicate, the mean value is displayed with error bar (± S.E). (**D**) The blots show the protein expression levels of p16, p21, p27, Cyclin D1, Cyclin D2, Ki-67 and γH2A.X in WI-38 fibroblast cell lines (subjected to different culture conditions of 100 or 150 days quiescence by contact inhibition and no quiescence induction) maintained in culture at 20% O_2_ until they approached senescence at late PD. The up or down-regulation was signified by the presence or absence of the bands in Western Blots. (**E, F, G, H, I, J, K**) Comparison of mean fold change of protein expression levels of p16 (E), p21 (F), p27 (G), Cyclin D1 (H), Cyclin D2 (I), Ki-67 (J) and γH2A.X (K) in WI-38 cell lines where quiescence was induced for 100 or 150 days by contact inhibition respectively compared to controls at corresponding span of time in culture. Cell lines were maintained at 20% O_2_ as triplicates. The bars indicate the mean ± S.D. ** p<0.01, *** p<0.001 - significantly different compared to fibroblasts with PD assigned 1. n = 3 specifies the number of samples except for γH2A.X (S3K Fig. where n = 2).
(TIF)

S4 Fig **Effect of long term quiescence induction (100 or 150 days) in WI-38 fibroblasts maintained at 3% O_2_.** (**A**) Growth curve of 3 independent WI-38 fibroblast cell lines (control with no quiescence induction, and cell lines where quiescence was induced for 100 or 150 days respectively by contact inhibition and then maintained in culture till they approached senescence) maintained in culture at 3% O_2_ as triplicates from an early PD until senescence at late PDs. Each growth curve is measured in triplicate. Data points of all measurements are displayed (not the mean). (**B & C**) Percentage of SA-β gal positive cells at different time points of their growth in culture in the control WI-38 fibroblast cell line and in the cell lines where quiescence was induced for 100 or 150 days respectively. S4B and S4C Figs. are plotted with PDs and days in the y-axis respectively. Each curve is measured in triplicate, the mean value is displayed with error bar (± S.E). (**D**) The blots show the protein expression levels of p16, p21, p27, Cyclin D1, Cyclin D2, Ki-67 and γH2A.X in WI-38 fibroblast cell lines (subjected to different culture conditions of 100 or 150 days quiescence by contact inhibition and no quiescence induction) maintained in culture at 3% O_2_ until they approached senescence at late PD. The up or down-regulation was signified by the presence or absence of the bands in Western Blots. (**E, F, G, H, I, J, K**) Comparison of mean fold change of protein expression levels of p16 (E), p21 (F), p27 (G), Cyclin D1 (H), Cyclin D2 (I), Ki-67 (J) and γH2A.X (K) in WI-38 cell lines where quiescence was induced for 100 or 150 days by contact inhibition respectively compared to controls at corresponding span of time in culture. Cell lines were maintained at 3% O_2_ as triplicates. The bars indicate the mean ± S.D. * p<0.05, ** p<0.01, *** p<0.001 - significantly different compared to fibroblasts with PD assigned 1. n = 3.
(TIF)

S5 Fig **Impact of Staurosporine treatment in young and old PD MRC-5 fibroblasts.** (**A**) Percentage of apoptotic cells in MRC-5 fibroblast cell lines (young PD = 34) treated with different concentrations of Staurosporine for different time spans (**B**) Percentage of SA-β gal positive cells in MRC-5 fibroblast cell lines (young PD = 34) treated with different concentrations of Staurosporine for different time spans (**C**) Percentage of apoptotic cells in MRC-5 fibroblast cell lines (old PD = 68) treated with different concentrations of Staurosporine for different time spans (**D**) Percentage of SA-β gal positive cells in MRC-5 fibroblast cell lines (old PD = 68) treated with different concentrations of Staurosporine for different time spans. In each instance, MRC-5 fibroblasts were maintained in culture at 20% O_2_ as triplicates. In A & C, the bars indicate the mean ± S.D. The mean values in B & D is displayed with error bar (± S.E). (**E**) The blots show the protein expression levels apoptotic protein Bax in MRC-5 fibroblast cell lines (young PD = 34 & old PD = 68) treated with 0.1 or 2.0 µM of Staurosporine for 96 hrs compared to controls (0 hrs). The MRC-5 fibroblasts were maintained in culture at 20% O_2_. The up or down-regulation was signified by the presence or absence of the bands in Western Blots. n = 3.
(TIF)

S6 Fig **Impact of Etoposide or Staurosporine treatment in MRC-5 fibroblasts subjected to short or long term quiescence.** (**A**) Percentage of apoptotic cells in MRC-5 fibroblast cell lines (untreated and treated with 7.5 µM Etoposide or 2.0 µM Staurosporine for 96 hrs) maintained at different culture conditions - after 9 (MRC-5 PD 35) or 150 days (MRC-5 PD 32) of quiescence induction by contact inhibition, fibroblasts of young PD = 34 and in fibroblasts of old PD = 68. (**B**) The blots show the protein expression levels of apoptotic protein Bax in MRC-5 fibroblast cell lines (young PD = 34, MRC-5 cell lines subjected to 150 consecutive days of quiescence induction by contact inhibition) treated with 2.0 (Staurosporine) or 7.5 µM (Etoposide) of apoptotic agents compared to controls (0 hrs). The MRC-5 fibroblasts were maintained in culture at 20% O_2_. The up or down-regulation was signified by the presence or absence of the bands in Western Blots. Values statistically different from their controls (t-test) are indicated with an asterix: * p<0.05, ** p<0.01, *** p<0.001. n = 3.
(TIF)

S7 Fig **Intersection of the most commonly differentially regulated genes with age (both up and down) in IMR-90 fibroblasts subjected to replicative senescence** [97] **compared to genes retrieved from MRC-5 fibroblasts subjected to long term quiescence induction compared to their controls.** Venn plots of DEGs in young vs. senescent IMR-90 fibroblasts (red circles, data of [97]) and young (MRC-5, PD 32) vs. quiescent MRC-5 fibroblasts (PD 32+150 days quiescent; blue circles, data determined here), (**A**) up-, (**B**) down-regulated. Both numbers of DEGs in the intersection are significant with regards to the expected overlap of two independent groups.
(TIF)

S8 Fig **Heatmap showing scaled gene-set level changes for KEGG pathways identified using gene set enrichment analysis.** The left column represents the comparison of young and senescent IMR-90 cells [97] while the right column is based on expression changes between young and long-term quiescent MRC-5 fibroblasts. Each KEGG pathway was found to be significantly regulated in IMR-90, MRC-5 or both datasets. The histogram on the left explains the color coding (e.g. red boxes encode down-regulation in senescent IMR-90 and quiescent MRC-5 fibroblasts).
(TIF)
