# Supplementary figures and images for: Long-Term Quiescent Fibroblast Cells Transit into Senescence
Source: PLoS One. 2014 Dec 22;9(12):e115597. doi: 10.1371/journal.pone.0115597 (PMC4274099; doi:10.1371/journal.pone.0115597)

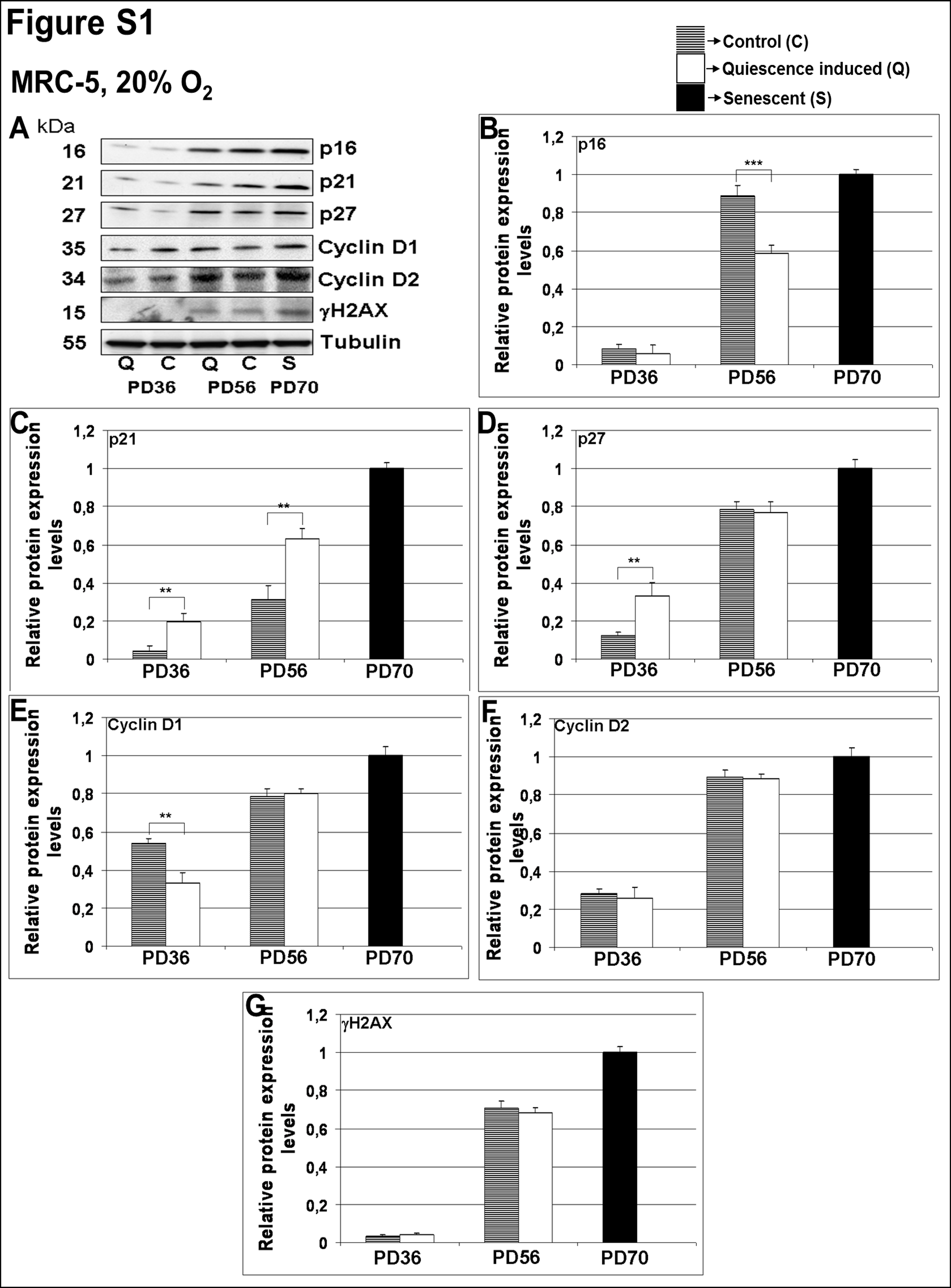

Supplement: S1 Fig — Effect of short term quiescence induction on protein expression levels of a number of cell cycle associated genes and a marker for DNA damage in MRC-5 fibroblasts. (A) The blots show the protein expression levels of p16, p21, p27, Cyclin D1, Cyclin D2 and γH2A.X in two MRC-5 fibroblast cell lines (control with no quiescence induction and a cell line where quiescence was induced 3 times separately for a span of 9 days) maintained at 20% O2 at different stages of their span in culture. The up or down-regulation was signified by the presence or absence of the bands in Western Blots. (B, C, D, E, F, G) Comparison of mean fold change of protein expression levels of p16 (B), p21 (C), p27 (D), Cyclin D1 (E), Cyclin D2 (F) and γH2A.X (G) in 3 times quiescence induced MRC-5 cell lines and control MRC-5 cell lines maintained in culture as triplicates. The bars indicate the mean ± S.D. ** p<0.01, *** p<0.001 - significantly different compared to fibroblasts with PD assigned 1. n = 3. (TIF) [file pone.0115597.s001.tif]

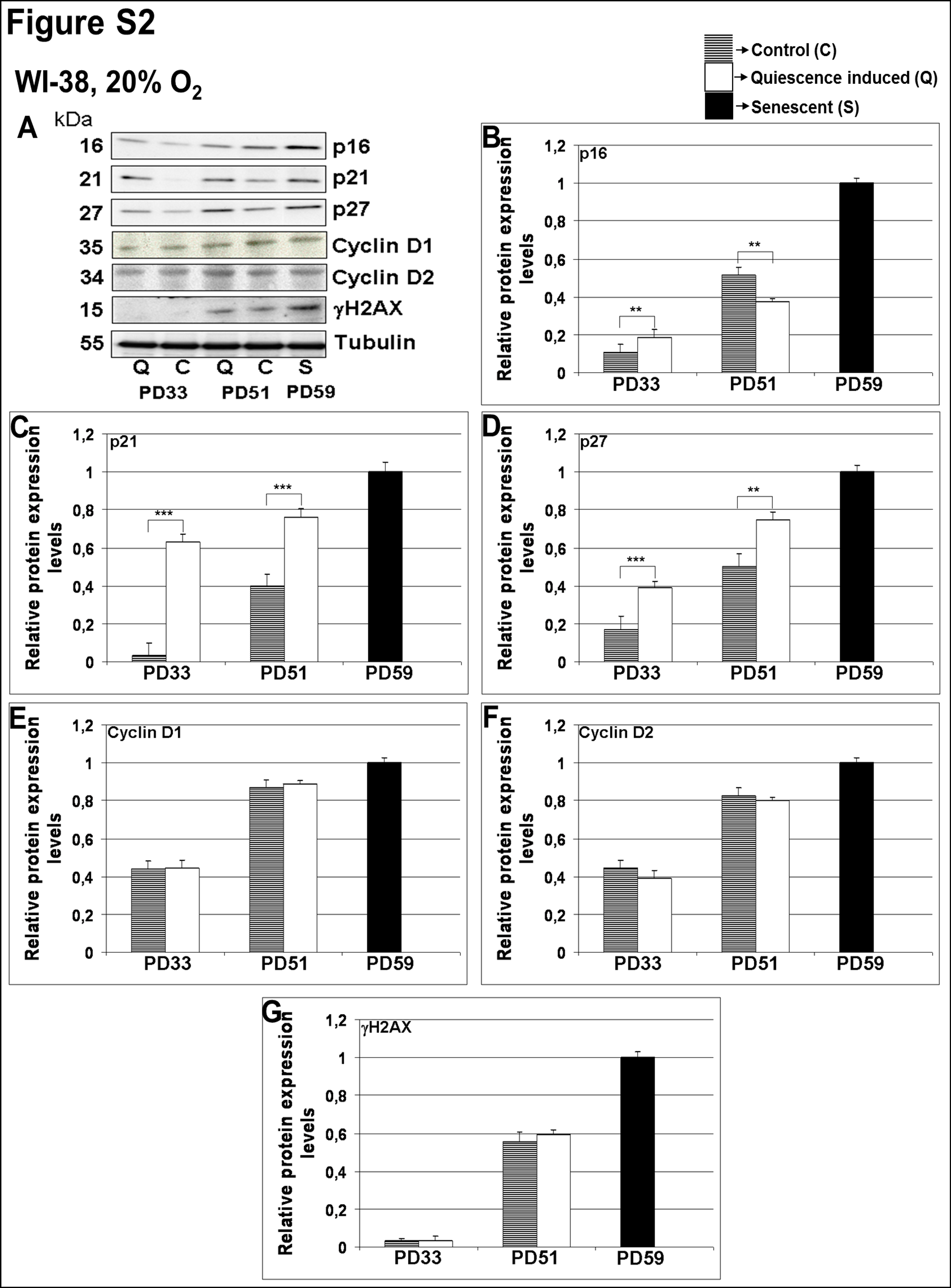

Supplement: S2 Fig — Effect of short term quiescence induction on protein expression levels of a number of cell cycle associated genes and a marker for DNA damage in WI-38 fibroblasts. (A) The blots show the protein expression levels of p16, p21, p27, Cyclin D1, Cyclin D2 and γH2A.X in two WI-38 fibroblast cell lines (control with no quiescence induction and a cell line where quiescence was induced 3 times separately for a span of 9 days) maintained at 20% O2 at different stages of their span in culture. The up or down-regulation was signified by the presence or absence of the bands in Western Blots. (B, C, D, E, F, G) Comparison of mean fold change of protein expression levels of p16 (B), p21 (C), p27 (D), Cyclin D1 (E), Cyclin D2 (F) and γH2A.X (G) in 3 times quiescence induced WI-38 cell lines and control WI-38 cell lines maintained in culture as triplicates. The bars indicate the mean ± S.D. ** p<0.01, *** p<0.001 - significantly different compared to fibroblasts with PD assigned 1. n = 3. (TIF) [file pone.0115597.s002.tif]

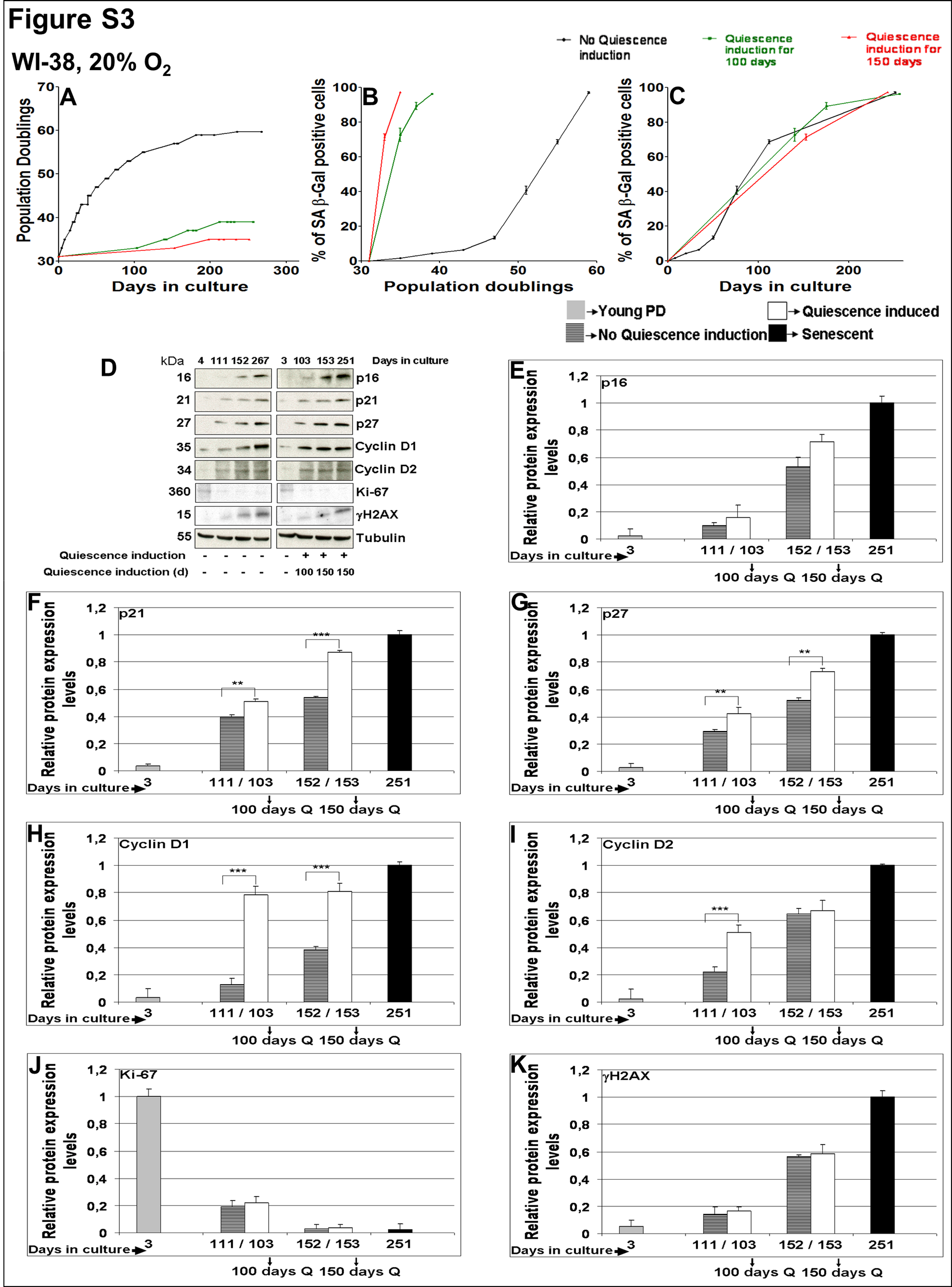

Supplement: S3 Fig — Effect of long term quiescence induction (100 or 150 days) in WI-38 fibroblasts maintained at 20% O2. (A) Growth curve of 3 independent WI-38 fibroblast cell lines (control with no quiescence induction, and cell lines where quiescence was induced for 100 or 150 days respectively by contact inhibition and then maintained in culture till they approached senescence) maintained in culture at 20% O2 as triplicates from an early PD until senescence at late PDs. Each growth curve is measured in triplicate. Data points of all measurements are displayed (not the mean). (B & C) Percentage of SA-β gal positive cells at different time points of their growth in culture in the control WI-38 fibroblast cell line and in the cell lines where quiescence was induced for 100 or 150 days respectively. S3B and S3C Figs. are plotted with PDs and days in the y-axis respectively. Each curve is measured in triplicate, the mean value is displayed with error bar (± S.E). (D) The blots show the protein expression levels of p16, p21, p27, Cyclin D1, Cyclin D2, Ki-67 and γH2A.X in WI-38 fibroblast cell lines (subjected to different culture conditions of 100 or 150 days quiescence by contact inhibition and no quiescence induction) maintained in culture at 20% O2 until they approached senescence at late PD. The up or down-regulation was signified by the presence or absence of the bands in Western Blots. (E, F, G, H, I, J, K) Comparison of mean fold change of protein expression levels of p16 (E), p21 (F), p27 (G), Cyclin D1 (H), Cyclin D2 (I), Ki-67 (J) and γH2A.X (K) in WI-38 cell lines where quiescence was induced for 100 or 150 days by contact inhibition respectively compared to controls at corresponding span of time in culture. Cell lines were maintained at 20% O2 as triplicates. The bars indicate the mean ± S.D. ** p<0.01, *** p<0.001 - significantly different compared to fibroblasts with PD assigned 1. n = 3 specifies the number of samples except for γH2A.X (S3K Fig. where n = 2). (TIF) [file pone.0115597.s003.tif]

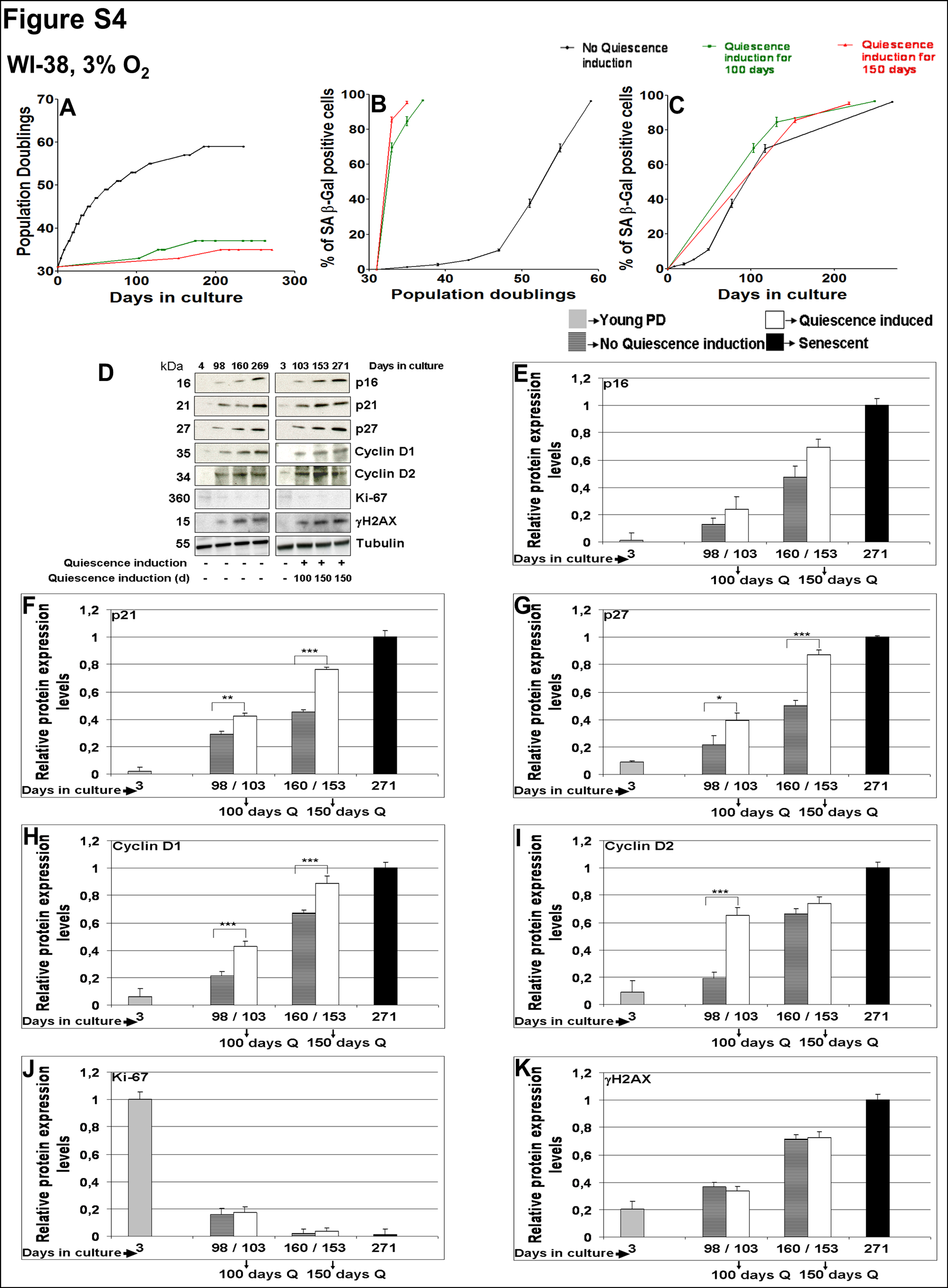

Supplement: S4 Fig — Effect of long term quiescence induction (100 or 150 days) in WI-38 fibroblasts maintained at 3% O2. (A) Growth curve of 3 independent WI-38 fibroblast cell lines (control with no quiescence induction, and cell lines where quiescence was induced for 100 or 150 days respectively by contact inhibition and then maintained in culture till they approached senescence) maintained in culture at 3% O2 as triplicates from an early PD until senescence at late PDs. Each growth curve is measured in triplicate. Data points of all measurements are displayed (not the mean). (B & C) Percentage of SA-β gal positive cells at different time points of their growth in culture in the control WI-38 fibroblast cell line and in the cell lines where quiescence was induced for 100 or 150 days respectively. S4B and S4C Figs. are plotted with PDs and days in the y-axis respectively. Each curve is measured in triplicate, the mean value is displayed with error bar (± S.E). (D) The blots show the protein expression levels of p16, p21, p27, Cyclin D1, Cyclin D2, Ki-67 and γH2A.X in WI-38 fibroblast cell lines (subjected to different culture conditions of 100 or 150 days quiescence by contact inhibition and no quiescence induction) maintained in culture at 3% O2 until they approached senescence at late PD. The up or down-regulation was signified by the presence or absence of the bands in Western Blots. (E, F, G, H, I, J, K) Comparison of mean fold change of protein expression levels of p16 (E), p21 (F), p27 (G), Cyclin D1 (H), Cyclin D2 (I), Ki-67 (J) and γH2A.X (K) in WI-38 cell lines where quiescence was induced for 100 or 150 days by contact inhibition respectively compared to controls at corresponding span of time in culture. Cell lines were maintained at 3% O2 as triplicates. The bars indicate the mean ± S.D. * p<0.05, ** p<0.01, *** p<0.001 - significantly different compared to fibroblasts with PD assigned 1. n = 3. (TIF) [file pone.0115597.s004.tif]

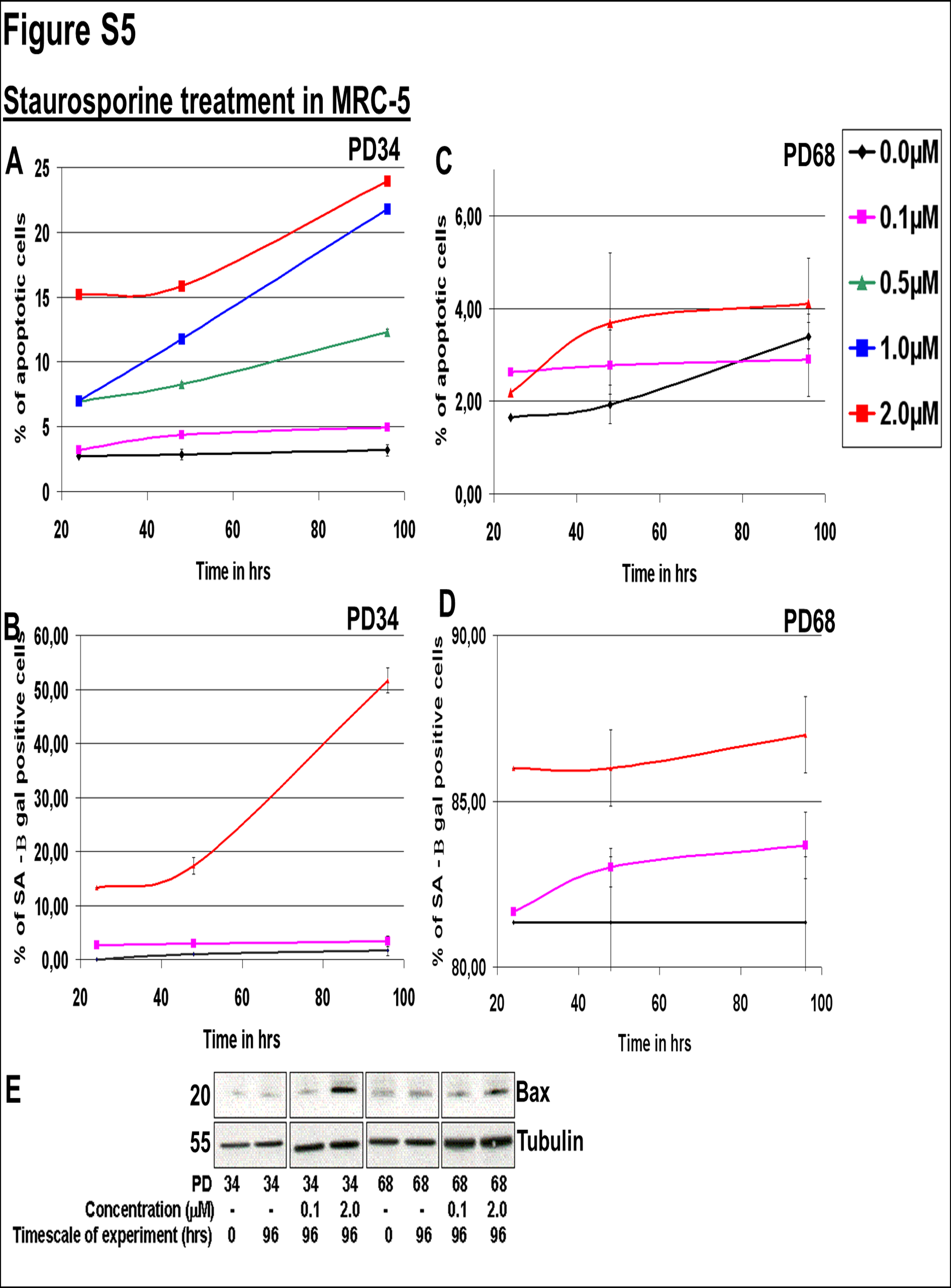

Supplement: S5 Fig — Impact of Staurosporine treatment in young and old PD MRC-5 fibroblasts. (A) Percentage of apoptotic cells in MRC-5 fibroblast cell lines (young PD = 34) treated with different concentrations of Staurosporine for different time spans (B) Percentage of SA-β gal positive cells in MRC-5 fibroblast cell lines (young PD = 34) treated with different concentrations of Staurosporine for different time spans (C) Percentage of apoptotic cells in MRC-5 fibroblast cell lines (old PD = 68) treated with different concentrations of Staurosporine for different time spans (D) Percentage of SA-β gal positive cells in MRC-5 fibroblast cell lines (old PD = 68) treated with different concentrations of Staurosporine for different time spans. In each instance, MRC-5 fibroblasts were maintained in culture at 20% O2 as triplicates. In A & C, the bars indicate the mean ± S.D. The mean values in B & D is displayed with error bar (± S.E). (E) The blots show the protein expression levels apoptotic protein Bax in MRC-5 fibroblast cell lines (young PD = 34 & old PD = 68) treated with 0.1 or 2.0 µM of Staurosporine for 96 hrs compared to controls (0 hrs). The MRC-5 fibroblasts were maintained in culture at 20% O2. The up or down-regulation was signified by the presence or absence of the bands in Western Blots. n = 3. (TIF) [file pone.0115597.s005.tif]

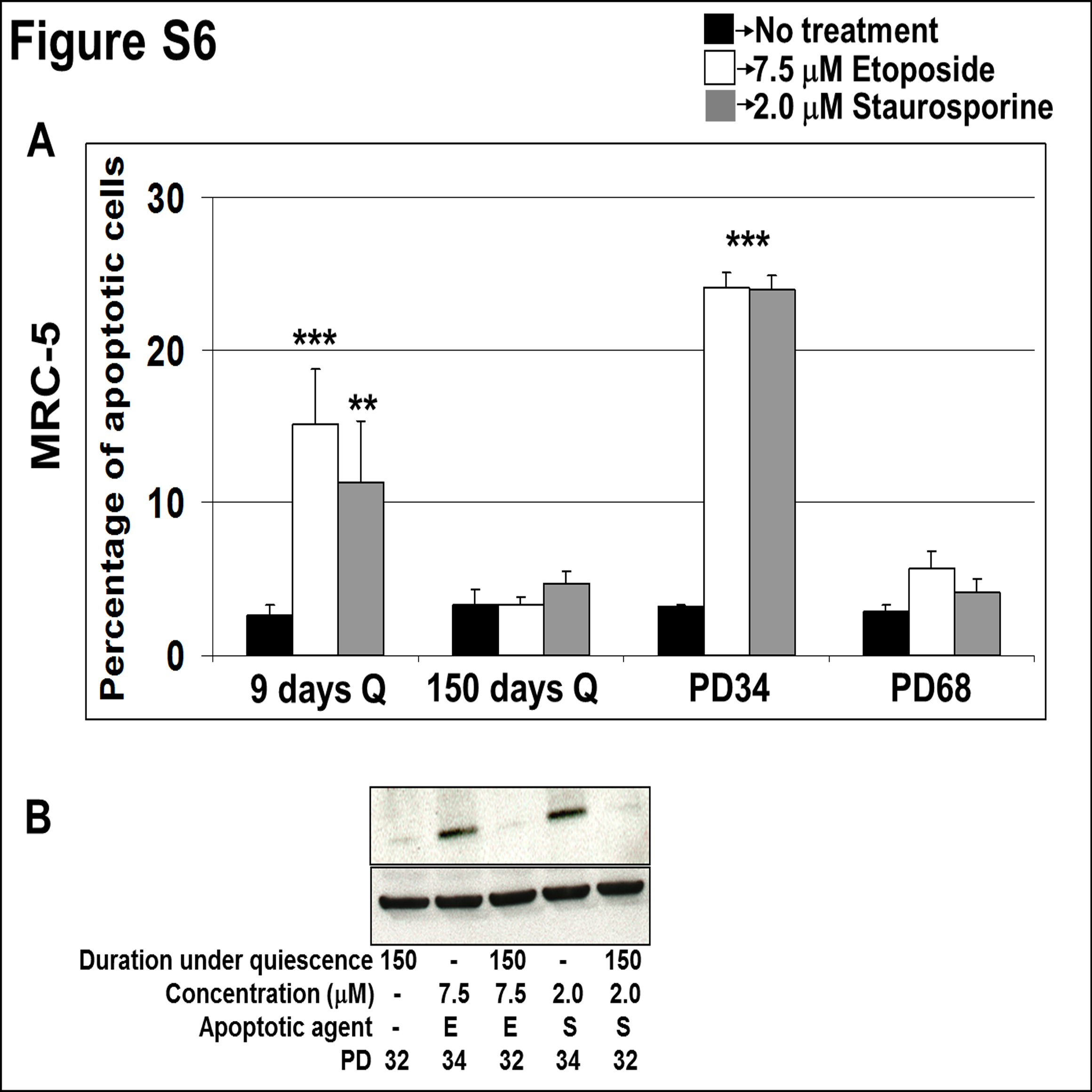

Supplement: S6 Fig — Impact of Etoposide or Staurosporine treatment in MRC-5 fibroblasts subjected to short or long term quiescence. (A) Percentage of apoptotic cells in MRC-5 fibroblast cell lines (untreated and treated with 7.5 µM Etoposide or 2.0 µM Staurosporine for 96 hrs) maintained at different culture conditions - after 9 (MRC-5 PD 35) or 150 days (MRC-5 PD 32) of quiescence induction by contact inhibition, fibroblasts of young PD = 34 and in fibroblasts of old PD = 68. (B) The blots show the protein expression levels of apoptotic protein Bax in MRC-5 fibroblast cell lines (young PD = 34, MRC-5 cell lines subjected to 150 consecutive days of quiescence induction by contact inhibition) treated with 2.0 (Staurosporine) or 7.5 µM (Etoposide) of apoptotic agents compared to controls (0 hrs). The MRC-5 fibroblasts were maintained in culture at 20% O2. The up or down-regulation was signified by the presence or absence of the bands in Western Blots. Values statistically different from their controls (t-test) are indicated with an asterix: * p<0.05, ** p<0.01, *** p<0.001. n = 3. (TIF) [file pone.0115597.s006.tif]

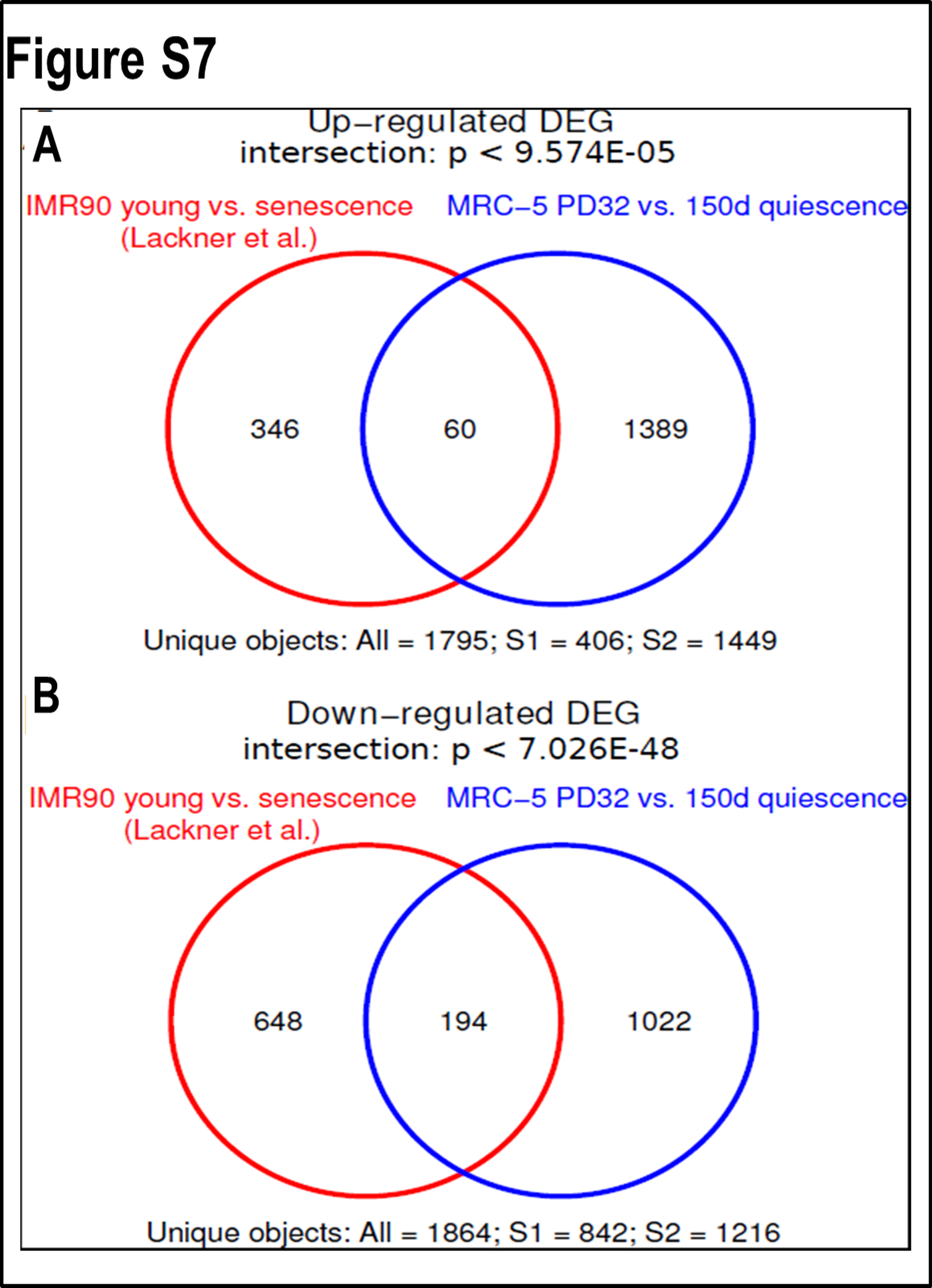

Supplement: S7 Fig — Intersection of the most commonly differentially regulated genes with age (both up and down) in IMR-90 fibroblasts subjected to replicative senescence [97] compared to genes retrieved from MRC-5 fibroblasts subjected to long term quiescence induction compared to their controls. Venn plots of DEGs in young vs. senescent IMR-90 fibroblasts (red circles, data of [97]) and young (MRC-5, PD 32) vs. quiescent MRC-5 fibroblasts (PD 32+150 days quiescent; blue circles, data determined here), (A) up-, (B) down-regulated. Both numbers of DEGs in the intersection are significant with regards to the expected overlap of two independent groups. (TIF) [file pone.0115597.s007.tif]

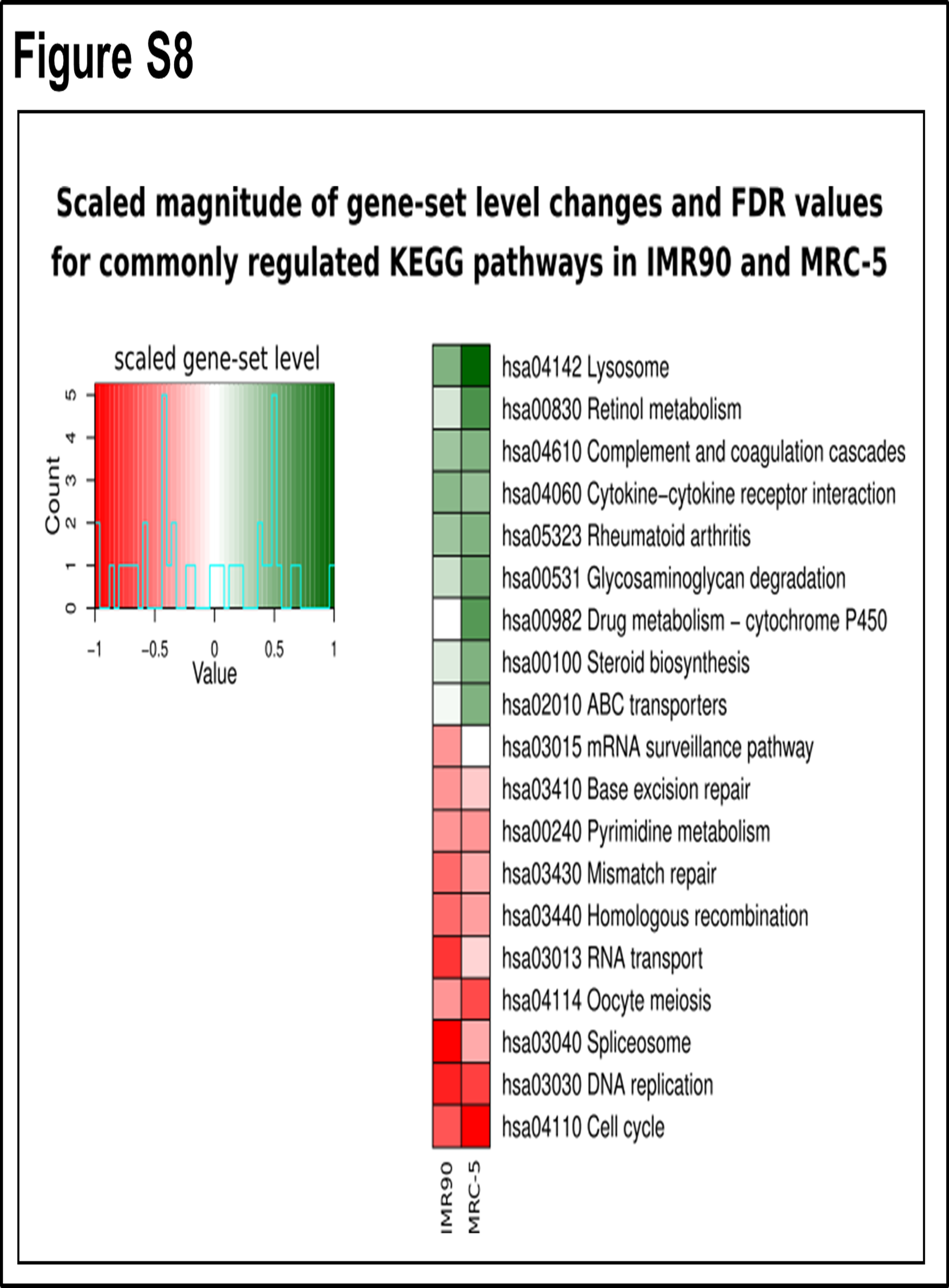

Supplement: S8 Fig — Heatmap showing scaled gene-set level changes for KEGG pathways identified using gene set enrichment analysis. The left column represents the comparison of young and senescent IMR-90 cells [97] while the right column is based on expression changes between young and long-term quiescent MRC-5 fibroblasts. Each KEGG pathway was found to be significantly regulated in IMR-90, MRC-5 or both datasets. The histogram on the left explains the color coding (e.g. red boxes encode down-regulation in senescent IMR-90 and quiescent MRC-5 fibroblasts). (TIF) [file pone.0115597.s008.tif]
